# How Cancer Online Support Groups Work, for Whom, and in What Circumstances: Realist Review

**DOI:** 10.2196/77445

**Published:** 2026-05-13

**Authors:** Jacqueline L Bender, Sarah Scruton, Geoff Wong, Lauren R Squires, Stephanie Babinski, Marina Englesakis, Alaina Cyr, Henry Potts, Andrea Tricco, Richard Wassersug, Mary Jane Esplen, Victoria J Forster, Olga Perski, Holly O Witteman, Charlene Soobiah, Janet Papadakos, Colleen Young, Colleen Fox

**Affiliations:** 1 Cancer Rehabilitation and Survivorship, Department of Supportive Care, Princess Margaret Cancer Centre, University Health Network, 585 University Avenue, Toronto, ON, M5G 2N2, Canada, 1 416-581-8606; 2 Dalla Lana School of Public Health, University of Toronto, Toronto, ON, Canada; 3 Nuffield Department of Primary Care Health Sciences, University of Oxford, Oxford, United Kingdom; 4 Library and Information Services, University Health Network, Toronto, ON, Canada; 5 Patient Education and Engagement, University Health Network, Toronto, ON, Canada; 6 Centre for Health Informatics and Multiprofessional Education, University College London, London, United Kingdom; 7 Li Ka Shing Knowledge Institute, St.Michael's Hospital, Unity Health Toronto, Toronto, ON, Canada; 8 Department of Cellular and Physiological Sciences, University of British Columbia, Vancouver, BC, Canada; 9 Department of Psychiatry, University of Toronto, Toronto, ON, Canada; 10 Women's College Hospital, Toronto, ON, Canada; 11 Faculty of Social Sciences and Herbet Wertheim School of Public Health, Tampere University, Tampere, Finland; 12 Human Longevity Science, University of California, San Diego, San Diego, CA, United States; 13 Faculté de médicine, Université Laval, Quebec City, QC, Canada; 14 Cancer Education Program, University Health Network, Toronto, ON, Canada; 15 Health Education and Content Services, Mayo Clinic Hospital, Rochester, MN, United States; 16 Ontario Health, Toronto, ON, Canada

**Keywords:** online support group, online community, realist review, systematic review, cancer

## Abstract

**Background:**

Online support groups (OSGs) offer a convenient way for patients with cancer and survivors of cancer to obtain information and support. However, not all OSGs are helpful, and in some cases, they may increase distress. Overall, there is a lack of clear evidence on the effects of OSGs, along with how and why they work.

**Objective:**

This study examined how, for whom, and in what contexts OSGs work for patients with cancer and survivors of cancer.

**Methods:**

A realist review of the evidence on cancer OSGs was conducted (12 databases, inception to February 15, 2025). We followed Pawson’s steps and the RAMESES (Realist and Meta-Narrative Evidence Syntheses: Evolving Standards) quality standards for realist reviews. The Mixed-Methods Appraisal Tool was used to assess the quality of the evidence. Using a realist logic of analysis, we generated a narrative summary of the findings, context-mechanism-outcome configurations, and a program theory (eg, conceptual framework) to explain how cancer OSGs work. Additionally, we developed evidence-based recommendations for optimizing the effectiveness of OSGs.

**Results:**

Of 16,659 papers identified, 168 were included. The evidence was organized into 3 categories, 10 concepts, and 57 context-mechanism-outcome configurations. Cancer OSGs can support patients by providing informational, emotional, appraisal, and altruistic support. This can lead to changes in distress, isolation, empowerment, and self-esteem, through negative and positive appraisals as well as coping efforts. These outcomes, however, depend on user attitudes about OSGs (ie, trustworthy, useful, culturally safe, and easy-to-use), how well the OSG fits their needs (ie, health need, sociodemographic or clinical characteristics, and coping style), and perceptions of control (ie, availability, anonymity, privacy, and autonomy). If an OSG was a good fit for a user’s needs, whether they experienced positive outcomes depended on features of the communication technology (ie, modality, response time, and visual social cues), group composition and dynamics (ie, norms, moderation, safety, cohesion, and belonging), and the nature and content of interactions (ie, emotional expression, cognitive processing, and empathetic responses). These factors trigger underlying mechanisms such as feeling understood, accepted, cared for, valued, reassured, informed, and confident, which result in positive outcomes.

**Conclusions:**

OSGs can address supportive care needs and improve psychosocial well-being for patients with cancer and survivors of cancer. However, outcomes depend on specific contexts and mechanisms that impact how well OSGs meet patients’ needs. To optimize effectiveness of OSGs, we recommend (1) helping to assess fit and address specific needs; (2) demonstrating trustworthiness; (3) enhancing anonymity and control, and protecting privacy; (4) enhancing ease-of-use; (5) supporting connection and belonging; (6) encouraging activity; (7) enhancing the nature of content shared to boost therapeutic effects; and (8) monitoring and adjusting design and management strategies.

## Introduction

With close to 20 million diagnoses and 9.7 million deaths, cancer is the leading noninfectious disease worldwide [1]. By 2050, the number of new cases of cancer is expected to reach 35 million, and over two-thirds of people diagnosed with cancer will become long-term survivors [1]. Patients with cancer and survivors of cancer face multiple physical, functional, and psychosocial challenges during and after cancer treatment [2-7] that affect their quality of life [8-10], resulting in a high burden of supportive care needs [4,11-14].

As many as 1 in 4 patients with cancer and survivors of cancer may turn to online support groups (OSGs) for peer support to address their needs [15]. OSGs, also known as online communities, are virtual spaces where people with common conditions come together to obtain and give information and support [16]. These virtual spaces—ranging from email lists, discussion forums, or groups on social media platforms such as Facebook [17], X (formerly Twitter) [18], and Reddit [19]—may be moderated by professionals or peers [20]. Frequently reported advantages of OSGs include their anonymity, followed by their availability, similarity, and diversity of experiences, and greater information and resources [21].

Several studies suggest that OSGs reduce feelings of isolation, depression, and anxiety, and enhance knowledge, coping, empowerment, and self-management among patients with cancer and survivors of cancer [22-28]. However, OSGs are not risk-free, and in some cases, may increase distress. A randomized controlled trial of an unmoderated OSG for newly diagnosed patients with breast cancer found that participants in the intervention group did worse over time on measures of distress and quality of life compared to controls [29]. Yet 60% of intervention participants reported high satisfaction. These conflicting findings suggest that the benefits of OSGs may depend on individual and contextual factors.

Overall, systematic reviews of the effectiveness of cancer OSGs have found a heterogeneous evidence base of positive, negative, and null findings, and few have examined their underlying mechanisms [20,30-32]. To enhance their effectiveness, there is a need to better understand how, why, for whom, and in what contexts cancer OSGs work. A realist review of the evidence [33] can address these questions and has been successfully used to inform the optimization of other complex interventions [34-37]. Therefore, this study aimed to conduct a realist review of the evidence to understand how, why, for whom, and in what contexts OSGs work for patients with cancer and survivors of cancer.

## Methods

### Study Design

We undertook a realist review [33] of the evidence on cancer OSGs. Realist reviews aim to build and test theories that explain how complex interventions work by examining how underlying causal processes (eg, mechanisms) in specific circumstances (eg, contexts) interact to produce results (eg, outcomes) [38]. We followed the steps by Pawson et al [33] and the RAMESES (Realist and Meta-Narrative Evidence Syntheses: Evolving Standards) quality standards for realist reviews (Checklist 1) [38]. Our methods were described in detail in a published protocol [39] that was registered on PROSPERO (International Prospective Register of Systematic Review; #CRD42021250046).

### Knowledge User Involvement

An integrated knowledge translation approach [40] was followed. Five categories of knowledge users (n=15 in total) were engaged in the project: (1) program developers who have experience developing OSGs; (2) community strategists and moderators that manage and moderate OSGs; (3) clinicians who refer patients to or deliver OSGs; (4) patients, survivors, and family members who have used or created their own “grassroots” OSGs; and (5) health care administrators and policymakers responsible for implementing OSGs and driving best practices. Knowledge users were consulted when designing the study protocol, developing the initial program theory, refining the program theory, and generating best practice recommendations, and they participated as coauthors in the writing of this paper.

### Step 1: Initial Program Theory

An initial program theory was developed based on Bender’s multitheory framework on the use and effects of breast cancer OSGs [28], and a 2-hour consultation workshop with project team members and knowledge users. This multitheory framework draws on the Transactional Theory of Stress and Coping [41], Social Comparison Theory [42], the Technology Acceptance Model (TAM) [43], and the Theory of Planned Behavior [44]. As shown in our protocol paper [39], it suggests that people with cancer use OSGs to obtain information and emotional support from similar others to lessen negative appraisals of events, enhance feelings of control, and develop coping strategies, which in turn reduce or buffer anxiety. Beliefs about the usefulness and trustworthiness of OSGs influence adoption and engagement. The availability, anonymity, and low commitment afforded by the online medium are believed to enhance feelings of control. However, the effects of making social comparisons in OSGs may be positive or negative depending on the circumstances and the person’s goals.

### Step 2: Search for Evidence

The search strategies were developed by an information specialist (ME) and peer-reviewed by an external medical information professional following the PRESS (Peer Review of Electronic Search Strategy) guidelines [45]. Given the range of terms for OSGs and cancer diagnoses, our search strategy was intentionally broad, opting for sensitivity over precision (Multimedia Appendix 1). The following databases were searched from inception to February 15, 2025: MEDLINE, Embase, the Cochrane Database of Systematic Reviews, the Cochrane Central Register of Controlled Trials, Ovid EmCare Nursing, APA PsycINFO (all via Ovid), CINAHL (Ebscohost), and Scopus (Elsevier). In addition, 2 clinical trial registries were searched (ClinicalTrials.gov and WHO ICTRP), along with dissertations (ProQuest Digital Dissertations International) and books or chapters (University of Toronto Summon OneSearch).

### Step 3: Paper Selection and Appraisal

The inclusion and exclusion criteria were based on a foundational systematic review of OSGs conducted by Eysenbach et al [20], with feedback from team members and knowledge users (Table 1). The papers gathered from the search were uploaded to Rayyan systematic review management software for screening and selection [46]. To assess interrater agreement, 4 researchers (SB, LRS, AC, and CS) independently screened the same 100 titles and abstracts using a pilot-tested screening tool (Table 2). As 90% agreement was established, the remaining records were divided among the 4 researchers to screen independently, with meetings held every 2 weeks to track progress and discuss and resolve uncertainties. A 10% random sample was audited by the lead author, and disagreements were resolved through discussion. The intentionally broad search strategy yielded a significant number of irrelevant papers (eg, negative answer to at least 1 screening question), the majority of which were excluded at the title and abstract screening stage.

**Table 1.** Inclusion and exclusion criteria.

Criteria	Inclusion	Exclusion
Population	People diagnosed with cancer and their family caregivers.	Neither includes people diagnosed with cancer and their family caregivers, nor does it include subgroup analysis of this group. Only includes family caregivers.
Intervention	An online support group (or an online intervention with an online support group component) is defined as a group of individuals with similar or common health-related interests who communicate through a communication platform on the internet.	Does not include an online support group, or does not include subgroup analysis of an online support group in a multicomponent intervention.
Study type	Any type of study design that investigates the factors that influence the use of online support groups or the effects of participation in online support groups.	Studies involving content analyses of communication occurring between members of an online support group that did not examine the perceived effect of participation in the online support group.
Outcomes	All types of outcomes associated with participation in online support groups, including but not limited to user engagement, knowledge, self-efficacy, social support, psychological, behavioral, and physiological outcomes, or use of health services.	Outcomes unrelated to participation in online support groups.
Publication	Papers, including peer-reviewed primary research papers, secondary research papers (eg, reviews), books, or commentaries published in English from database inception to February 15, 2025.	Papers not published in English and outside this time frame. Preprints were excluded from the search strategy. Conference abstracts, study protocols, and dissertations were excluded during paper selection.

**Table 2.** Paper screening tool.

Question	Result
Are any of the participants people diagnosed with cancer or their caregivers?	Yes ⇨no exclusion
Is the intervention or program online, or does it contain an online component?	Yes ⇨no exclusion
Does the intervention include an online support group? (Including, eg, a discussion forum, live chat, email list, online community, peer-to-peer communication, or social media like Twitter [X Corp], Facebook [Meta], Instagram [Meta], Snapchat [Meta], and Reddit [Reddit Inc])	Yes ⇨no exclusion
Does the record describe factors that influence the use of OSGs or what the effects of participation (eg, lurking or posting) are (ie, social support, information, reduced distress, and reduced isolation)?	Yes ⇨no exclusion

The relevance and rigor of papers were assessed in 2 phases. Relevance was determined by assessing the extent to which the data could contribute to developing and testing context-mechanism-outcome configurations (CMOC) [33]. This was conducted by 3 team members (SB, LRS, and SS). A total of 2 team members independently assessed the relevance of each paper that passed the title and abstract screening and met to resolve uncertainties. A 10% random sample was audited by the lead author, and disagreements were resolved through discussion. Rigor was determined postanalysis for CMOCs with fewer than 5 supporting papers. This approach is recommended for realist reviews when there is sufficient data to develop and test CMOCs, as was the case for most CMOCs in this study [47]. Rigor was determined by assessing whether the methods were credible and trustworthy using the Mixed-Method Appraisal Tool [48]. To establish interrater agreement, 3 team members (SS, LRS, and JLB) independently assessed the same 5 papers using the Mixed-Method Appraisal Tool. Each remaining paper was independently assessed by 2 team members, who met to resolve uncertainties. The results of the rigor assessment are shown in Tables3  4.

**Table 3.** MMAT^a^ quality assessment results.

Qualitative	Item number						
	S1	S2	1.1	1.2	1.3	1.4	1.5
Sullivan [49], 2003	Yes	Yes	Yes	Yes	Yes	Yes	Yes
Bender et al [28], 2013	Yes	Yes	Yes	Yes	Yes	Yes	Yes
Griffiths et al [50], 2015	Yes	Yes	Yes	Yes	Yes	Yes	Yes
Sandaunet [51], 2008	Yes	Yes	Yes	Yes	Cannot tell	Yes	Yes
Im et al [52], 2007	Yes	Yes	Yes	Yes	Yes	Yes	Yes
Vilhauer et al [53], 2011	Yes	Yes	Yes	Yes	Yes	Yes	Yes
Chiu and Hiseh [54], 2013	Yes	Yes	Yes	Yes	Yes	Yes	Yes
Donovan et al [55], 2019	Yes	Yes	Yes	Yes	Yes	Yes	Yes
Shaw et al [56], 2000	Yes	Yes	Yes	Yes	Yes	Yes	Yes
Stephen et al [57], 2014	Yes	Yes	Yes	Yes	Yes	Yes	Yes
van Uden Kraan et al [24], 2008	Yes	Yes	Yes	Yes	Yes	Yes	Yes
Vilhauer [58], 2014	Yes	Yes	Yes	Yes	Yes	Yes	Yes
Ure et al [59], 2020	Yes	Yes	Yes	Yes	Yes	Cannot tell	Yes
Owen et al [60], 2009	Yes	Yes	Yes	Yes	Yes	Yes	Yes
Hoybye et al [61], 2005	Yes	Yes	Yes	Yes	Cannot tell	Yes	Yes

a MMAT: Mixed-Method Appraisal Tool.

**Table 4.** MMAT^a^ quality assessment results.

Quantitative descriptive	Item number						
	S1	S2	4.1	4.2	4.3	4.4	4.5
Yoo et al [62], 2013	Yes	Yes	Yes	Yes	Yes	N/A^b^	Yes
Bender et al [28], 2013	Yes	Yes	Yes	Yes	Yes	Yes	Yes
Leimeister et al [63], 2008	Yes	Yes	Yes	Yes	Yes	N/A	Yes
Malloch and Taylor [64], 2019	Yes	Yes	Yes	Yes	Yes	N/A	Yes
Cabling et al [65], 2018	Yes	Yes	Yes	Cannot tell	Yes	N/A	Yes
Lieberman et al [66], 2005	Yes	Yes	Yes	Cannot tell	Yes	N/A	Yes
Hoybye et al [67], 2010	Yes	Yes	Yes	Cannot tell	Yes	Yes	Yes
Owen et al [60], 2009	Yes	Yes	Yes	Yes	Yes	N/A	Yes
Donovan et al [55], 2019	Yes	Yes	Cannot tell	Yes	Yes	Cannot tell	Yes

a MMAT: Mixed-Method Appraisal Tool.

b N/A: not applicable.

Qualitative study criteria [48] asked the following question: (S1) Are there clear research questions? (S2) Do the collected data allow for addressing the research question? (1.1) Is the qualitative approach appropriate to answer the research question? (1.2) Are the qualitative data collection methods adequate to address the research question? (1.3) Are the findings adequately derived from the data? (1.4) Is the interpretation of results sufficiently substantiated by data? (1.5) Is there coherence between qualitative data sources, collection, analysis, and interpretation?

Quantitative descriptive study criteria [48] included the following questions: (S1) Are there clear research questions? (S2) Do the collected data allow for addressing the research question? (4.1) Is the sampling strategy relevant to address the research question? (4.2) Is the sample representative of the target population? (4.3) Are the measurements appropriate? (4.4) Is the risk of nonresponse bias low? (4.5) Is the statistical analysis appropriate to answer the research question?

Quantitative nonrandomized study criteria [48] included the following questions: (3.1) Are the participants representative of the target population? (3.2) Are measurements appropriate regarding both the outcome and the intervention (or exposure)? (3.3) Are there complete outcome data? (3.4) Are the confounders accounted for in the design and analysis? (3.5) During the study period, was the intervention administered (or exposure occurring) as intended?

Quantitative nonrandomized study criteria [48] included the following questions: (S1) Are there clear research questions? (S2) Do the collected data allow for addressing the research question? (3.1) Are the participants representative of the target population? (3.2) Are measurements appropriate regarding both the outcome and the intervention (or exposure)? (3.3) Are there complete outcome data? (3.4) Are the confounders accounted for in the design and analysis? (3.5) During the study period, was the intervention administered (or exposure occurred) as intended?

### Step 4: Extracting and Organizing the Data

Two team members (SB and LRS) extracted the study characteristics from each paper using a pilot-tested study extraction tool. Patient demographics (Multimedia Appendix 2 [22-24,28,29,49-210]) and intervention characteristics (Multimedia Appendix 3 [22-24,28,29,49-210]) were extracted. In parallel, 1 team member (SB) uploaded the papers into NVivo (version 12; Lumivero) qualitative analysis software to code sections of text that were relevant to supporting, refining, or refuting aspects of the program theory. A preliminary coding framework was developed based on the concepts in the initial program theory. A total of 2 researchers (SB and LRS) used the coding framework to code a random sample of 5 papers to determine agreement and refine the coding framework, and then 2 team members (SB and SS) coded each of the remaining papers. Coding consisted of initially grouping the data into high-level conceptual “buckets” that were defined iteratively through discussions with the lead author (JLB).

### Step 5: Synthesis and Analysis

In the final step, data sorted into conceptual buckets were analyzed to determine whether the data were functioning as context, mechanisms, or outcomes [33]. This analysis was undertaken by 2 team members (JLB and SS) trained in realist methodology guided by an expert in realist methodology (GW). Further, 1 team member (SS) identified prominent patterns of contexts and outcomes in the data and sought to explain them through mechanisms by which they occurred, generating CMOCs. The findings were iteratively confirmed, refuted, or refined, first by JLB and then by GW through discussion with SS. The findings were shared with this project’s team and knowledge users for their feedback in a final 2-hour consultation workshop.

## Results

### Study Characteristics

**Figure 1.**
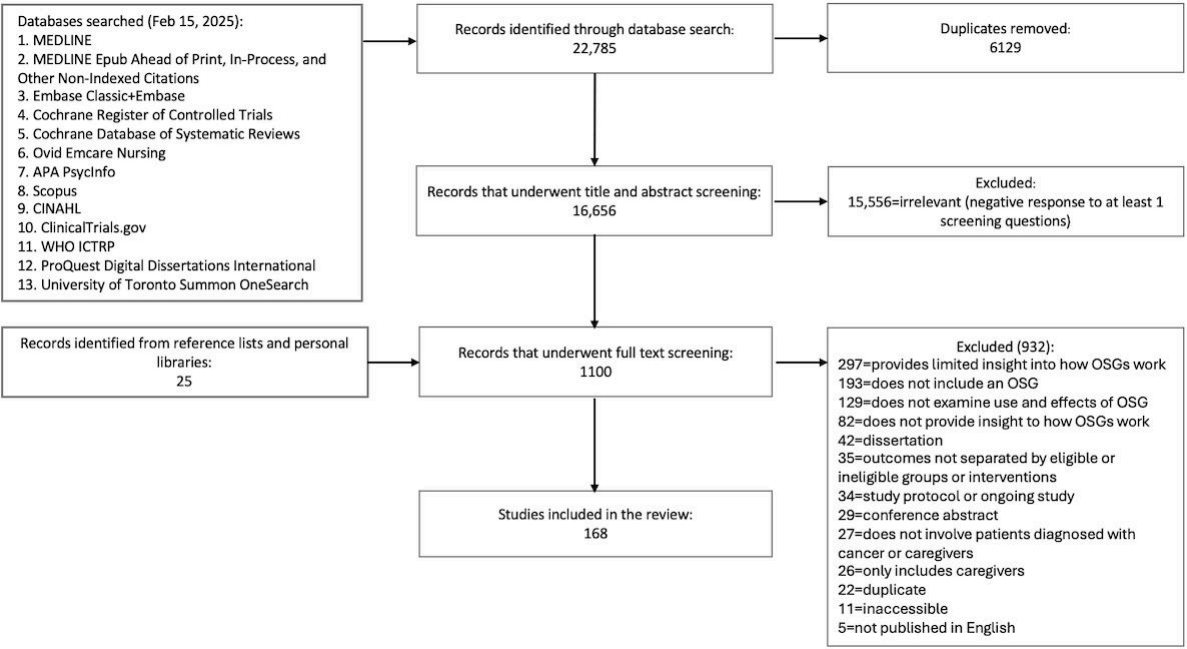
Flow diagram. OSG: online support group; WHO: World Health Organization.

A total of 168 papers were included in the analysis, which involved a range of 1 (case study) to 180,000 (content analysis) study participants (Figure 1). Studies were conducted within a span of 25 years (1999‐2025). See Table 5 and MultimediaAppendices 2  3 for further details.

**Table 5.** Study characteristics (n=168).

Characteristic	Values, n (%)
Country (of first author)	
United States	109 (64.9)
United Kingdom	16 (9.5)
Australia	12 (7.1)
Netherlands	9 (5.4)
Canada	8 (4.8)
Other (Denmark, Germany, Norway, Taiwan, South Korea, Japan, Finland, and China)	14 (8.3)
Study type	
Content analyses	39 (22.6)
Mixed methods	33 (11.9)
Quantitative (cross-sectional surveys)	25 (16.7)
Qualitative (interviews or focus groups)	26 (16.7)
Randomized controlled trials	14 (12.5)
Reviews	11 (6.5)
Commentaries	7 (3.6)
Pretest and posttest measurement	4 (6)
Case studies	3 (1.2)
Quantitative (longitudinal surveys)	3 (1.2)
Multiple methods	3 (1.2)
Cancer types	
Breast	71 (42.3)
Prostate	7 (4.2)
Ovarian	3 (1.8)
Other (eg, thyroid, colorectal, hematological, lung, sarcomas, testicular, head, and neck)	11 (6.5)
Multiple cancer types	50 (29.8)
Not specified	26 (15.5)
Sex	
All female	68 (40.5)
All male	10 (6)
Majority female (>50%)	50 (29.8)
Majority male (>50%)	6 (3.6)
Equal male + female	1 (0.6)
Not specified	33 (19.6)
Race or ethnicity	
Not specified	101 (60.1)
Majority White (>50%)	51 (30.4)
Majority non-White	16 (9.5)
Education levels	
Not described	89 (53)
The majority (>50%) postsecondary education (ie, some college or higher)	76 (45.2)
50% or more without postsecondary education	3 (1.8)
Technology type	
Multicomponent websites (eg, a website with multiple support services, such as information, decision support, and peer support; may include different OSG^a^ types)	44 (26.2)
Bulletin board or discussion forum	37 (22)
Not specified (eg, studies of people who use OSGs)	32 (19)
Other (eg, newsgroups, live chats, mailing lists, social networking sites, and video chats)	26 (15.5)
Multiple types (ie, multiple OSGs in 1 study)	29 (17.3)
Synchronous vs asynchronous	
Asynchronous only	97 (57.7)
Synchronous only	6 (3.6)
Not specified	44 (26.2)
Both	21 (12.5)
Moderator present	
Yes	41 (24.4)
No	8 (4.8)
Not specified	99 (58.9)
Multiple OSGs with one or more moderators	20 (11.9)
Level of moderation description	
Not described	109 (64.9)
High (eg, described moderators and the moderation strategy)	24 (14.3)
Low (eg, limited description of moderator and moderation strategy)	35 (20.8)
Moderator type	
Trained professional, study staff, or organization staff	20 (11.9)
HCP^b^	11 (6.5)
Lay peer	4 (2.4)
Trained peer	1 (0.6)
Combination	5 (3)
Multiple OSGs with one or more having an HCP or professional moderator	6 (3.6)
Not described	121 (72)

a OSG: online support group.

b HCP: health care provider.

### Main Findings

#### Overview

**Figure 2.**
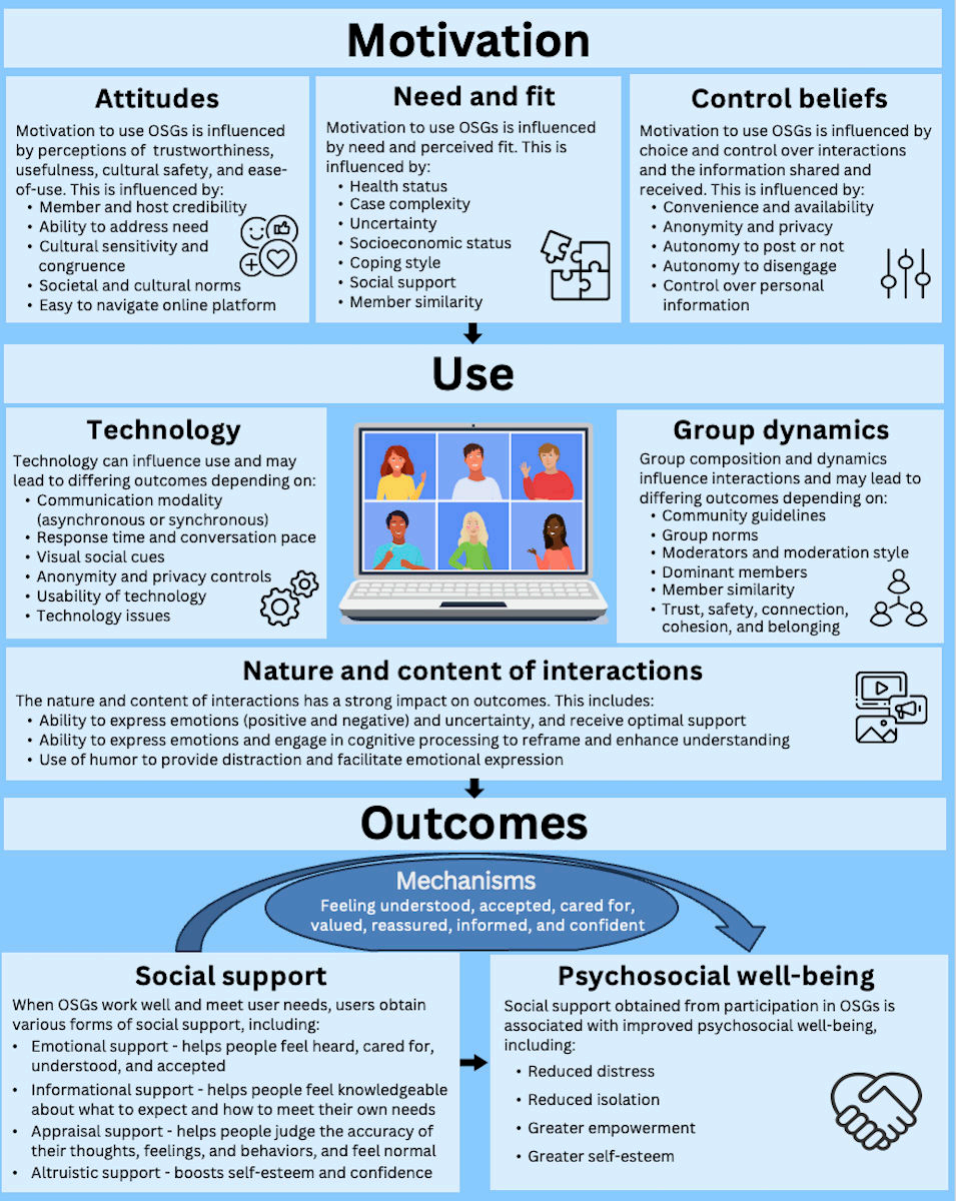
Refined program theory. *Mechanisms leading to positive outcomes

The evidence was organized into 3 overarching categories and 10 concepts: motivation (attitudes, need or fit, and control beliefs), use (communication technology factors, group composition and dynamics, and nature and content of interactions), and outcomes (informational, emotional, validatory, and altruistic support). Additionally, a total of 57 CMOCs were generated. CMOCs are labeled (eg, A1, B1, etc) and described in the subsequent paragraphs. The papers that contributed to each CMOC are found in Table 6. A visual depiction of the refined program theory is provided in Figure 2.

**Table 6.** Categories, concepts, and CMOCs^a^.

ID code^b^	CMOC	Paper reference
Motivation		
Attitudes		
A1	When an individual cannot assess the trustworthiness and intentions of other group members (C^c^), they will be less likely to use OSGs^d^ (O^e^) because they are not certain about their credibility (M^f^).	[23,68-72]
A2	When individuals join OSGs that are more relevant to their cancer journey stage (C), they are more likely to use them or benefit (O) because their questions or concerns will be addressed or it will meet their needs (O).	[24,28,50,51,73-79]
A3	When an individual does not have a timely need for information or support (C), they may reduce or stop their use of an OSG (O), because the OSG is no longer useful to them (M).	[28,51,80,81]
A4	When an individual is not sure if a particular OSG will meet their expectations and needs (C), they may join many groups and lurk in them (O) because they want to learn more about the group before engaging (M).	[28,58,69,71,82,83]
A5	When an individual’s culture stigmatizes cancer (C), they may be less likely to use OSGs (O) because they may feel uncomfortable disclosing their cancer to others for fear of stigma (M).	[52,70,84-86]
A6	When OSGs are not culturally sensitive (C), individuals may be less likely to use them (O) because they may feel less comfortable participating in them (M).	[70,84,85,87-89]
A7	When OSGs are not easy to use (C), individuals will be less likely to use them (O) because it makes them feel frustrated (M).	[28,50,58,74,90,91]
Need or fit		
B1	When an individual has a complex case or needs (C), they may be more likely to use OSGs (O) because they are distressed and therefore need more support from individuals in a similar situation (M).	[28,53,69,70,76,81,92-105]
B2	When individuals do not have strong offline support systems (C), they may be more likely to use OSGs (O) because they have more of a need for them (M).	[68,86,94,95,97,105-118]
B3	When individuals feel that the informational and emotional support provided by their health care providers is not sufficient (C), they will be more likely to use OSGs (O) because it is a convenient way to access the support they want from other people who have experienced cancer (M).	[24,28,54,55,80,85,94,108,110,111,114,117,119-125]
B4	When patients with cancer do not want to burden their family and friends about their illness (C), they may be more likely to use OSGs (O) because OSGs can provide an alternative avenue for support (M).	[24,49,56,57,59,81,86,118,121,123,125-128]
B5	When trusted or credible others (eg, health professionals or friends) do not trust OSGs (C), individuals may be less likely to use them (O) because they value the opinion of the trusted other (M).	[23,28,68,129,130]
B6	When an individual has low eHealth literacy (C), they may be less likely to use OSGs (O) because they are not comfortable with the technology (M).	[23,62,79,95,131-133]
B7	When individuals face fewer barriers to accessing, understanding, or using OSGs (C), they may be more likely to use OSGs (O) because of ease of use (and convenience; M).	[67,68,70,76,77,95,96,115,131,134-140]
B8	People with emotion-focused coping styles (C) may be more likely to use OSGs or more likely to benefit (O) because they feel safety or anonymity facilitates emotional expression (M).	[62,67,106,107,141,142]
B9	People with avoidant coping styles (C) may be less likely to use OSGs or less likely to benefit (O) because they may feel distressed by others expressing their emotions in the OSGs (M).	[62,67,106,107]
B10	When an individual reads distressing information in an OSG (C), they may reduce or stop their use of an OSG (O) to avoid feeling distressed by the information (M).	[28,51,80,101,143-145]
Control beliefs		
C1	When peer support is provided online (C), users can access the support more easily (O) because it removes the barriers that individuals often face when accessing in-person support (M).	[49,56-58,60,87,91,105,108,110,111,114,117,137,146-149]
C2	The 24/7 availability of OSGs (C) makes people feel better supported (O) because they can have their needs met at any time (M).	[28,50,56,58,60,69,148,150]
C3	Users can access OSGs to look for information that they need, whenever they need it (C), which makes users feel more in control (O) because they can manage the type of information they receive and when they receive it (M).	[49,58,59]
C4	When OSGs are perceived by potential users as having privacy issues (C), individuals will be less likely to use them (O), because they are concerned their personal information will be leaked or used without their permission (M).	[23,28,52,69,71,136,151,152]
Use		
Communication technology factors		
D1	When people using OSGs take the time to reflect on what they want to write and how to write it (C), the relevance and content of the posts improve (O) because they can carefully craft a thoughtful post or response (M).	[56,57,72]
D2	When individuals with a pressing concern post in OSGs and there is a delay in response (or no response; C), it can make users feel isolated (O) because they are led to believe that their concerns are unimportant or unworthy of a response (M).	[56,61,69,150,153]
D3	When the posts and responses in an OSG are fast-paced (eg, in a live chat; C), those seeking support may not find this helpful (O), because they find the discussion hard to follow, overwhelming, or lacking in depth (M).	[56,57,90,101]
D4	Text-based only communication in OSGs (C) can make it harder for users to feel connected (O) because of the lack of visual cues (M).	[57,60,63,87,154]
D5	Text-based only communication in OSGs (C) can cause miscommunication (O) because of the lack of visual cues (M).	[60,61,63,101]
D6	When OSGs allow users to use different modes of interaction (ie, emojis, reactions, and likes; C), it makes it easier for users to interpret posts (O) because it makes up for the lack of visual and audio cues (M).	[60,71,150,153,155,156]
D7	When OSGs include the option to have personalized profiles that include personal information about them, their interests, and their particular circumstances (C), it makes it easier for users to feel connected to one another (O), because the information shared in profiles helps users get to know and relate to each other (M).	[50,90,135,153,157]
D8	When OSGs are used in conjunction with in-person support groups (C), users receive greater social support (O) because they receive the unique benefits of both types of support (M).	[28,55]
D9	When OSGs offer some privacy and anonymity (C), users feel more comfortable disclosing information and emotions (O) because they feel safer and less likely to be embarrassed or shamed (M).	[22,23,28,50,56,57,60,61,70,72,81,84,86,126,129,147,148,151,158]
D10	When users are provided the option of having private discussions with individuals or subgroups of individuals (C), it makes them feel more comfortable disclosing information and emotions (O) because they value the privacy this provides (M).	[50,59,121,133,157,159]
Group composition and dynamics		
E1	When OSG members adhere to the group’s norms (C), they are likely to benefit (O) because they feel accepted by the group (M).	[64,71,94,146,160-164]
E2	When an OSG has a social norm that discourages discussion of negative topics (C), it can make users feel isolated (O) because they cannot receive needed support about that topic (M).	[51,53,58,65,71,81,101,126,154]
E3	When OSGs have moderators who are active, encouraging, and supportive (C), users will be more likely to actively participate (O) because moderators facilitate conversations (M).	[50,55,57,58,74,90,101,165,166]
E4	When moderators provide emotional support to OSG members (C), users will be more likely to actively participate in OSGs (O) because they feel supported (M).	[50,66]
E5	When OSGs have knowledgeable moderators who share resources and correct misinformation (C), users are more likely to trust the information shared in the OSG (O) because they believe the OSG is trustworthy or credible (M).	[50,51,70,72,88,90,144,148,152]
E6	When an OSG has one or more dominant members (ie, moderators and informal group members) who overstep by posting and behaving in a negative way (C), other group members feel uncomfortable (O) because they judge these acts to be inappropriate (M).	[52,65,69]
E7	When OSGs are structured in a way that facilitates the formation of close bonds or connections (ie, smaller groups, similarities between group members, supportive dynamic, and humor; C), users are more likely to benefit (O) because group members feel better supported (M).	[49,53,56-60,66,68,69,72,101,121-123,127,144,153-155,157,164,167-170]
E8	When OSGs enable users to connect with people in similar situations whom they do not know offline (C), users feel less isolated (O) because they learn that there are others like them (M).	[50-53,56,59,60,69,71,81,82,94,119,121,125,126,148,167,171-174]
E9	When an OSG provides a psychologically safe space where members can trust each other (C), users feel more comfortable disclosing information and emotions (O) because they do not have to be concerned about negative repercussions (M).	[71,157,175-177]
E10	When an individual has a poor prognosis (C), they may feel less comfortable disclosing information and emotions (especially mixed stage or cancer type groups; O) because they do not want to “scare” or distress other users (M).	[53,64,65,101]
E11	When an individual feels that their thoughts and experiences are different from the group (C), they may reduce or stop their use of an OSG (O) because they are concerned that they will not be accepted (M).	[22,51,53,65,144]
Nature and content of interactions		
F1	When OSG users express negative emotions and thoughts in an OSG (C), they experience stress relief (O) because writing about a stressful event allows one to process and learn from one’s emotions (M).	[49,50,54,56,57,60,83,99,101,106,107,119,121,122,126-128,150,153,154,156,172,178-181]
F2	When an OSG member makes a post that includes emotional expression and uncertainty (C), they are more likely to receive a response (O) because other users recognize that they need support (M).	[81,120,146,172,180,182-185]
F3	When individuals discuss their cancer experience with other members of an OSG (C), it can help them cope with their cancer diagnosis (O) because it helps them to cognitively process and make sense of their illness (M).	[22,24,49,53,54,61,74,81,92,126,127,142,150,167,186-194]
F4	When people who use OSGs dislike opening up emotionally and creating emotional bonds (C), they are more likely to provide mainly informational support (O) because this is what they feel comfortable doing (M).	[49,71,126,134,136,154,158,195]
F5	When OSG members use appropriate humor to interact with each other (C), they are more likely to benefit (O) because humor serves as a distraction and breaks the tension or provides relief for stressful situations (coping mechanism; M).	[49,68,71,126,153,154]
F6	When individuals actively participate in an OSG by frequently posting and commenting (C), they receive more support from others (O) because their requests for support receive more responses (M).	[29,63,75,77,93,95,123,139,155,186,188,196-200]
F7	When individuals lurk and read posts in OSGs (C), they can still benefit (C), because they can obtain informational and emotional support from reading others’ posts (M).	[23,77,83,94,101,123,127,129,156,157]
Outcomes		
Social support		
G1	When an OSG has members who are willing to share and help interpret information (C), users gain knowledge (O), and their uncertainty is reduced (O) because they have a better understanding of their disease and what to expect (M).	[22-24,28,49,50,52,56,59,61,69,70,72-74,79,81,82,101,119,120,122-125,127-129,131,139,140,144,146,148,150,153-157,162,167,172,173,177,193,201-205]
G2	When individuals receive advice and encouragement from other OSG users on how to improve their care experience (C), they become more actively involved in their care (O) because they feel more empowered (M).	[22,24,49,101,122-124,144,154,157,193,201,206]
G3	When an OSG member receives supportive comments (eg, empathetic, compassionate, encouragement, and/or expressions of solidarity responses; C), they feel emotionally supported (O), because they know other people care about them (M).	[24,32,50,52,54-56,59,71,73,74,79,101,112,120-123,125,127,128,146,154-157,161,167,168,172,175,177,180,187,188,204,205,207,208]
G4	When individuals communicate with others in OSGs who have had similar experiences (C), they feel less isolated (O) because they receive validation that what they are going through is normal (M).	[24,49,50,55,56,78,79,82,101,126-128,150,201,205]
G5	When individuals who are stable or doing well read about the negative outcomes of other members of the OSG (C), they feel better about themselves (O) because they know they are doing better than other members (M).	[24,54,56,101]
G6	When individuals read about the positive outcomes of other members of the OSG (C), they feel hopeful or optimistic about their future (O) because other people have survived the disease, and that makes them feel that they could survive the disease as well (M).	[24,28,53,54,56,126,129]
G7	When individuals use OSGs to support others (C), it makes them feel good about themselves (O) because they are helping others (M).	[24,50,54,56,61,68,80,125,139,175,193]
G8	When individuals use OSGs to support others (C), it can help them to cope with their own cancer diagnosis (O) because it enables them to process and positively reframe their own experiences (M).	[24,61,75,77,78,93,109,128,139,178,182,188,209,210]

a CMOC: context-mechanism-outcome configuration.

b Codes refer to which categories, concepts, and context-mechanism-outcome configuration relate to respective themes throughout the paper.

c C: context.

d OSG: online support group.

e O: outcome.

f M: mechanism.

#### Motivation

Whether an individual chooses to use an OSG depends on their attitudes about OSGs, whether an OSG is a good fit for their needs and their control beliefs.

##### Attitudes

OSGs must meet certain criteria to be considered helpful. They must be trustworthy, useful, culturally congruent (eg, aligned with cultural norms) and culturally sensitive (eg, demonstrating knowledge, awareness, and acceptance of different cultures, and recognition of interpersonal power differentials [211]), and easy-to-use.

The trustworthiness of the OSG depends on how credible its members (eg, content and behaviors) and host organization (eg, community procedures and safeguards) appear. If either appears to be untrustworthy, people may avoid or leave the OSG due to concerns about receiving inaccurate information and misuse of their personal information, leading to a privacy breach (A1).

The usefulness of OSGs depends on how well their content matches an individual’s needs. Those recently diagnosed, deciding on treatment, or experiencing treatment side effects may benefit more from OSGs because they have needs that are likely to be discussed (A2). Conversely, individuals with fewer needs, such as those farther from diagnosis, or with no evidence of disease, may stop using OSGs when they no longer need them (A3). Most users will explore or “shop around” OSGs to find the best fit for their needs (A4).

Cultural norms and the level of cultural sensitivity, congruence, and safety in OSGs may affect participation. Some individuals may be reluctant to join OSGs if their culture stigmatizes cancer or discourages the disclosure of personal information (A5). Others may prefer to rely on family or their religious community for support if an OSG lacks culturally relevant information and safety (A6). Representation across ethnicity, language, and cultural values can enhance perceived safety and encourage engagement.

Finally, even if an OSG meets these criteria, individuals may be unlikely to take part in it if it is not easy to use. Confusing or difficult-to-navigate online environments can lead to frustration and discourage OSG use (A7).

##### Need and Perceived Fit

Needs drive use. People experiencing uncertainty or complex care needs (ie, newly diagnosed or metastatic or advanced cancer) are more likely to seek support from OSGs because of distress, fear of recurrence, persistent or late treatment effects, or disease progression (B1). Individuals who lack sufficient offline support often have a greater need for support from OSGs (B2 and B3). This was particularly apparent during the COVID-19 pandemic, which led to an increase in OSG use as individuals were unable to access in-person support from cancer peers, family or friends. Some individuals with strong offline support turn to OSGs because they do not want to burden or distress their family and friends with cancer-related concerns (B4). Others may not use OSGs if their offline support networks question their safety and trustworthiness (B5).

Socioeconomic status and coping style may affect OSG use (B6 to B10). A broad range of sociodemographic (eg, age, sex, race, ethnicity, sexual orientation, etc) and clinical factors (eg, diagnosis, stage, issue, etc) may determine fit (E7). Lower socioeconomic status (ie, income or educational attainment) may limit access to OSGs due to lack of stable housing, internet access, or e-health literacy [212]; B6 and B7). Individuals with emotion-focused coping styles may be more likely to use OSGs as they facilitate emotional disclosure (B8). On the other hand, people with avoidant coping styles may find the emotional nature of posts in OSGs distressing and may withdraw from OSGs or avoid them altogether (B9 and B10). Additionally, finding similar others with shared experiences (in terms of sociodemographic and clinical characteristics) is important for relatability, sense of belonging, and perceived fit.

##### Control Beliefs

An individual’s sense of choice and control over their interactions, and the information they share and receive in OSGs, is a critical factor in determining use of OSGs. The online medium overcomes practical barriers to accessing support, such as time and distance, and affords individuals the convenience and control of accessing support safely, including during infectious disease outbreaks such as the COVID-19 pandemic (C1). In OSGs with members from diverse time zones, users are likely to receive a timely response, meaning that they can obtain support when needed (C2). Users can also limit the information they are exposed to in OSGs by not responding to or not reading posts, or by disengaging from a post, thread, or user that is distressing. Taking action to avoid distressing information in OSGs can prevent feelings of being overwhelmed or distressed, which may be more difficult in in-person groups (C3). However, users may experience a lack of perceived control when there is a lack of anonymity, and there are privacy concerns. For example, some individuals are hesitant to use OSGs—especially on public platforms (ie, Facebook)—where their personal information could be used or disclosed without their permission, which they fear could impact their personal relationships or health care (C4).

### Use

Once an individual finds an OSG that is a good fit, whether they experience positive outcomes depends on how they participate, the people involved, and the discussion that occurs. This is influenced by communication technology factors, group composition and dynamics (including the presence and nature of moderation), and the nature and content of interactions. These factors trigger underlying mechanisms such as feeling understood, accepted, cared for, valued, reassured, informed, and confident, which result in positive outcomes.

#### Communication Technology Factors

Communication modality, which influences user response time and the pace of the conversation, as well as technology issues in OSGs, impacts user experience. Asynchronous communication may improve the quality of support received as members can take the time to craft thoughtful messages and reflect on responses (D1). However, on some asynchronous platforms, posts that go unanswered may become buried and never receive a response, which can lead to feelings of being unsupported, isolated, and rejected (D2). Synchronous chats offer immediate responses, but the discussion is often fast and difficult to follow and may be hindered by technology issues (ie, poor Wi-Fi connection), which can overwhelm users and limit the ability to have deep, meaningful conversations (D3).

The lack of visual social cues in OSGs is commonly cited as a reason why OSGs may not fully replace in-person support. Visual social cues are important for connecting on an emotional level: feeling seen and heard, interpreting communication, building trust, and preventing or resolving conflicts (D4 and D5). OSGs may compensate for the lack of visual cues through communication features, such as “liking” posts and emojis, which help users express emotions (D6). Customizable personal profiles (eg, profile pictures, status updates, videos, and interests) also help users get to know and connect with one another (D7). However, some users feel that OSGs work best when used in combination with in-person support groups to experience the benefits of both forms of social support (D8).

Anonymity and privacy in OSGs encourage users to open up and disclose sensitive or emotional information, which is critical for maximizing benefits. When OSGs offer some degree of anonymity and privacy, users feel more comfortable disclosing information because they feel safer and less likely to be embarrassed or shamed (D9). These features may be valuable for some men and other individuals who are uncomfortable disclosing health conditions and emotions. Additionally, some users may feel more comfortable disclosing information and sharing emotions in private conversations (eg, private direct messaging) or with subgroups of individuals in OSGs, rather than with the entire OSG (D10).

#### Group Composition and Dynamics

Within each OSG, there exists a set of rules or community guidelines, formal or informal, explicit or implicit, that establish group norms for acceptable behavior to foster group cohesion and belonging (E1). These rules or community guidelines often outline unacceptable behaviors (eg, flaming, bullying, or harassment), communication styles (eg, rude and disrespectful), and topics of conversation (eg, medical advice).

Sometimes, OSG members may collectively decide which topics are unacceptable, such as those seen as “negative,” including feelings of hopelessness, despair, depression, and death, and members may be discouraged from discussing them directly (eg, called out and removed) or indirectly (eg, avoidance or nonresponse). This can make individuals seeking support for these topics feel isolated (E2). This is more common in nonprofessionally moderated or unmoderated OSGs, which emphasize a positive attitude and outcome. In contrast, professionally moderated OSGs typically encourage the expression of both positive and negative emotions and experiences.

Moderators in OSGs encourage participation by welcoming new members, asking questions, starting conversations, and connecting users to relevant topics and other users while also providing emotional support (E3 and E4). Professional moderators (ie, health care providers, study staff, and host organization staff) can increase the perceived trustworthiness and safety of the group by preventing the spread of misinformation, managing difficult emotions, and addressing inappropriate behavior. Moderators who are trained in cultural sensitivity and competency can further strengthen these efforts by providing support that is tailored to users’ cultural and social contexts (E5). However, dominant members can disrupt the conversation (ie, arguing with people who disagree with them), which can cause discomfort (E6). Clear guidelines and effective moderation are important for group safety and minimizing conflict.

OSG users can form strong bonds with fellow group members, leading to connection, cohesion, and belonging. Strong bonds in OSGs are built on empathy, authenticity, mutual support, and universality (eg, a shared quality, interest, or experience). The strength of these bonds is influenced by the size of the group (eg, more opportunities to interact and get to know each other), group cohesion (eg, extent to which group members are attracted to the group and its goals and feel a sense of belonging and influence), and member similarities (eg, similar cancer, age, gender, ethnicity, sexual orientation, or issue; E7). For some, relationships formed with OSG members evolve into genuine friendships that are stronger than those in their offline network. Finding in-person support from others who are going through similar situations can be challenging, especially for those who live in rural areas or who have rare health conditions. OSGs help users overcome geographic barriers, enabling users to find and form connections with similar others from anywhere in the world (E8).

For users to feel comfortable expressing emotions and disclosing sensitive and potentially stigmatizing information in an OSG, they must also trust group members. The level of trust is often a direct result of the strength of the bonds between group members, formed through reciprocal kindness, empathy, support, and encouragement (E9). However, users with complex care needs or who have a poor prognosis may be reluctant to disclose persistent or worsening side effects because they do not want to distress others or experience rejection (E10). Ultimately, the discussion of negative emotions and experiences that are not accepted by the group may lead people to withdraw from an OSG because of a lack of belonging and acceptance (E11).

#### Nature and Content of Interactions

The quality of support that users receive in OSGs depends on the nature and content of interactions between group members. When groups encourage emotional expression, users can experience stress relief because writing about a stressful event helps users express and process their emotions, fears, or uncertainty (F1). When posts express emotions and uncertainty, users are more likely to receive empathy, support, and encouragement, because others recognize that they are in distress (F2).

Posts that describe experiences can facilitate active coping as users can read inspiring stories, learn practical tips, and engage in cognitive processing to make sense of their own condition (F3). Some users prefer to provide information rather than emotional support, which helps other users by enhancing their understanding, preparedness, and sense of control (F4). Additionally, humor in OSGs can foster a more lighthearted and relaxed atmosphere that can help to release tension, inspire hope, build rapport, and facilitate disclosure (F5). For some, humor serves as a coping mechanism and form of stress relief by distracting from and lightening the burden of the disease.

Active participation and engagement with OSGs, by posting or replying to others, leads to greater benefits and better outcomes (F6). However, people who only read and do not post in OSGs (eg, lurkers) can still benefit. Not only can they receive informational support, but they can also gain emotional support by forming emotional ties with others, by learning from and connecting with others’ emotions and experiences (F7).

### Outcomes

If an OSG is a good fit for an individual’s needs, and encourages supportive, inclusive discussion, users can experience various forms of social support: informational support (advice or guidance), emotional support (expressions of comfort, caring, and encouragement), appraisal support (help to assess the appropriateness or normativeness of thoughts, feelings, and experiences), and altruism-related esteem support (helping others with no expectation of reward). These types of support lead to positive or negative appraisals and coping mechanisms, which impact psychosocial well-being by affecting distress, isolation, self-esteem, and empowerment. Given that social support is a multidimensional construct, the same exchange in an OSG can yield multiple forms of social support, providing multiple benefits simultaneously.

#### Informational Support

Informational support is commonly given in OSGs by sharing personal cancer experiences and helpful resources. Reading about other people’s cancer experiences helps users better understand their own health condition and what to expect, reducing their uncertainty and increasing their sense of control (G1). When users provide informational support, they often share links and references to websites, research studies, conference summaries, etc. Increased knowledge about one’s health condition often motivates users to become more actively involved in treatment decision-making and symptom and side effect management. Users are encouraged to advocate for themselves and ensure that they are receiving the best care (G2).

#### Emotional Support

Emotional support is commonly given in OSGs by conveying empathy. Posts that express emotions and uncertainty and a need for support receive the most responses, most often from individuals who understand because they have had a similar experience. Emotional support often goes together with esteem support. For example, the most supportive responses are often from individuals who demonstrate empathy by validating the person’s feelings and experiences, disclosing similar feelings and experiences, and offering words of encouragement to help them overcome the issues they are facing. As a result, the recipient feels heard, understood, accepted, less alone, empowered, and more optimistic (G3).

#### Appraisal Support

Users can also receive appraisal support, through feedback, social comparision, and validation, by sharing and comparing thoughts, feelings, and experiences. When users make lateral comparisons by comparing themselves to others with similar experiences, they learn that what they are going through is normal and to be expected, and that they will be able to get through it. This helps users realize that their thoughts, feelings, and experiences are appropriate, reducing uncertainty and isolation (G4). Users can make other types of comparisons in OSGs. Downward comparisons with people who are doing worse can make users feel better about their own situation, which enhances self-esteem (G5). Upward comparisons with people who are doing better can offer inspiration, motivation, or hope for the future (G6).

#### Altruistic Support

Finally, OSG users can experience the benefits of altruism by helping others. They may transition from a support seeker to a support provider, and the feeling of helping others can boost their self-esteem (G7). Providing support to others in OSGs can also benefit the support provider by helping them to cognitively process and positively reframe their own experiences (G8).

### Recommendations

Based on the findings, we have generated evidence-based practice recommendations to optimize the use and effects of OSGs for patients with cancer and survivors of cancer (Textbox 1).

Textbox 1. **Recommendations to enhance the experience and outcomes of online support groups.**

Help assess fit and address specific needs

- Communicate why the online support group was created, for whom, and the intended benefits.
- Enable users to find relevant support to address specific needs by leveraging technology (eg, optimized search function, dedicated subforums to help users find the discussions and people that match their needs, etc).
- Guide users to more suitable forums or other online support groups as their needs change.

Demonstrate trustworthiness

- Establish and demonstrate community rules or norms for acceptable behavior to foster a safe and respectful online environment.
- Implement a moderation policy to ensure timely responses and manage inaccurate information and inappropriate behavior.
- Adopt transparent data policies and procedures to protect participant privacy.

Enhance anonymity and control, and protect privacy

- Provide users with privacy controls to tailor privacy settings to their preferences.
- Limit personal information requested (and shared) to protect user privacy.
- Enable users to use pseudonyms or avatars to enhance anonymity and facilitate disclosure.

Enhance ease of use

- Ensure the system is intuitive and easy-to-use, and adaptable to the evolving community usage.
- Provide easy-to-find instructions on how to use the system (eg, add to a profile page, post a comment, create a new topic or thread, etc).

Support connection and belonging

- Promote inclusion and acceptance of different backgrounds, identities, and abilities.
- Ensure users can find others who are similar to them (lateral comparisons).
- Ensure users can find and connect with other users who have experience (upward comparisons).
- Ensure there are spaces for users to connect and support each other at all stages of the cancer care continuum.

Encourage activity

- Employ trained moderators (lay or professional) to welcome members, establish etiquette, tone and style, foster discussion, connection, and matching, and implement community rules.
- Engage and train an active core group of members to facilitate and maintain activity (altruistic support).
- Implement alerts that are customizable to the stage of the community life cycle to notify users of relevant content in a timely way and avoid overwhelming users with too many notifications.

Enhance the nature of the content shared to boost therapeutic effects

- Encourage posts with emotional disclosure, information sharing, and cognitive processing.
- Offer user tips on how to participate and craft posts for maximum benefit.
- Use reactions (like, helpful, caring, etc) and allow emojis to help users convey meaning and emotion.

Monitor and adjust design and management strategies to optimize effectiveness

- Monitor activity and implement proven community management strategies to boost activity and supportive exchanges.
- Monitor usability and implement ongoing technology improvements to enhance optimal activity and supportive exchanges.
- Make use of available technology and training to enhance moderators’ ability to provide timely, personalized support.

## Discussion

### Summary

This is the first realist review of the evidence on OSGs. The findings show that the functional provisions of social support gained from cancer OSGs can lead to changes in psychosocial outcomes such as distress, isolation, empowerment, and self-esteem, through positive and negative appraisals and coping efforts. However, whether an individual chooses to use OSGs as a source of support depends on their attitudes about OSGs, how well the OSG fits them and their needs, and their perceptions of control. In addition, whether users experience positive or negative outcomes depends on specific mechanisms that occur within OSGs that influence their reactions and reasoning. These underlying mechanisms are influenced by contextual aspects of the communication technology, group composition and dynamics, and nature and content posted in OSGs. Overall, this study confirms the potential benefits and pitfalls of OSGs for patients with cancer and survivors of cancer. It also highlights the complexity of factors that influence whether an individual uses and benefits from an OSG. These findings validate and extend the multitheory framework proposed by Bender et al [28] on the use and effects of breast cancer OSGs, resulting in a refined program theory to explain how, why, for whom, and in what contexts OSGs work, visually depicted in Figure 2.

### What Influences Use of OSGs as a Coping Strategy?

Patients with cancer and survivors of cancer are driven to OSGs to address a specific need. Drawing on TAM [43], our findings demonstrate that the decision to use a cancer OSG is influenced by its perceived usefulness and ease of use. The TAM-3 [213] posits that perceived usefulness is determined by social influence (eg, subjective norm and image) and job performance characteristics (eg, job relevance, output quality, and tangible results), while perceived ease of use is determined by control beliefs (eg, computer self-efficacy, perceptions of external control, computer anxiety, and computer playfulness) and usability characteristics (eg, objective usability and perceived enjoyment).

Perceived usefulness is the strongest determinant of OSG use, shaped by the OSG’s ability to meet a specific need, its perceived trustworthiness (eg, quality, currency, and empathic nature of user-generated posts and moderation), and its cultural acceptability and sensitivity. Cross-sectional surveys across diverse patients with cancer and survivors of cancer show that unmet supportive care needs [15] and distress [214] predict OSG use. Qualitative research across diverse health-related OSGs has shown that once needs are met or if needs are not met, patients and survivors withdraw from OSGs [28,51,80], and as time passes, online help-seeking behaviors decrease [144]. As Massimi et al [80] have suggested, departure from an OSG is not necessarily a failing of the site’s design but potentially a logical reaction to changing life circumstances. OSGs must demonstrate cultural sensitivity and competence to be appealing, relevant, and effective for diverse patients [84]. The benefits of OSGs are enhanced when users share a common language, culture, and life experience, and when moderators are trained in cultural sensitivity and competency [85].

Perceived ease-of-use influences OSG adoption, but to a lesser extent than perceived usefulness. Control beliefs (including eHealth literacy [212]) and objective usability are important determinants of ease-of-use. For example, patients with cancer who were confident using the internet were 4 times more likely to use the internet and social media as a resource [214]. The anonymity and privacy of OSGs are frequently reported advantages that afford users control over their personal information and make users feel more comfortable sharing personal information [23,56]. Whereas privacy concerns contributed to a lack of control over personal information and discouraged participation in OSGs [151]. Furthermore, studies have shown that engagement declines and users leave when a site is frustrating to use [90]. For example, Holtz et al [91] found that conversion of in-person support groups to virtual (video-based) support groups was hindered by technology factors, including poor Wi-Fi connection, background noise, and users speaking over one another. Designing OSGs to ensure ease-of-use and protect privacy will enhance engagement and participation.

### How Do OSGs Work to Influence Health Outcomes?

Whether patients with cancer and survivors of cancer benefit from OSGs depends on the mechanisms that occur in OSGs that influence their reactions and reasoning. According to the Transactional Theory of Stress and Coping by Lazarus and Folkman [41], how people interpret and appraise events is critical in determining the events’ stressfulness. There are two types of threat appraisals: (1) primary, which involve judgments about whether the event is a threat; and (2) secondary, which involve evaluations of personal and social resources available to cope with the event. Support from peers who have experienced a similar stressor can lessen negative appraisals of events and promote active coping efforts, thereby reducing or buffering anxiety. Several studies in this review confirm that by participating in an OSG, people can learn that their feelings and experiences are normal and to be expected, thereby improving their appraisal of their situation. The validation provided by peers normalizes the experience and reduces the stress associated with the uncertainty of the condition and the stigma imposed by society. Further, people can obtain information on effective ways of coping, which enhances feelings of preparedness, control, and self-esteem.

However, the effects of social support in cancer OSGs will be greatest if the support provided meets an individual’s needs. Optimal Matching Theory [215] posits that the benefits and deleterious effects of social support are due to the matching (or mismatching) of support in different contexts. If the desired type of support is provided, it will enhance psychological adjustment and well-being. For example, in a study of survivors of breast cancer, both misalignment of support desired and received, and unwanted support were associated with poor adjustment [216]. In another study, OSG users who received unwanted support fared worse, which likely led to feelings of helplessness, negatively impacting self-esteem [217]. According to Lazarus and Folkman [41], problem-focused coping, such as seeking information, is most effective when the stressor is controllable, whereas emotion-focused coping is more useful when the situation is not. However, studies have shown that coping strategies that enhance perceptions of control contribute to psychological adjustment regardless of the controllability of the stressor [218]. Collectively, these findings indicate that patients with cancer may require different types of social support at different times and highlight the importance of matching the support provided in OSGs with individuals’ needs for maximum benefit.

Optimal Matching Theory [215] also suggests that aligning the profile of the support provider with that of the support seeker may enhance the effectiveness of the support provided. According to the Social Comparison Theory by Festinger [42], people seek similar others in times of stress and uncertainty to judge the appropriateness of their thoughts, feelings, and behaviors. Not finding similar others or not being accepted by the group can enhance feelings of deviance or uniqueness, worsening anxiety and feelings of isolation. These experiences can cause people to withdraw from OSGs [51,53]. Shared qualities and experiences facilitate mutual identification, disclosure, and belonging because peers understand one another in a way that other network members cannot. The challenge in creating OSGs based on the similarity principle is that users are likely to vary on a host of sociodemographic, clinical, and lifestyle dimensions. OSGs could be better designed to facilitate optimal matching by increasing the specificity of forums and connecting people with similar others with search function optimization. Moderators could also be trained to facilitate group cohesion (sense of belonging, influence, mutual support, and identification with other group members [101,219]), which has been associated with better outcomes in professionally led support groups [220].

The variable effects of OSGs are likely due in part to the negative effects of social comparisons. First, lack of positive upward comparisons (eg, learning what to expect, how to cope, and that it is possible to get better) can increase anxiety and decrease perceptions of control [221]. This is likely why participants in unmoderated breast cancer OSG in a study by Salzer [29] experienced an increase in distress: it was comprised of newly diagnosed patients who had no opportunity to make positive upward comparisons with survivors of cancer. Second, negative social comparisons (eg, learning that you are not doing as well as everyone else or that it is possible to get worse) can increase anxiety, decrease self-esteem, and decrease perceptions of control [221]. For example, being exposed to patients in a breast cancer OSG who were doing poorly was anxiety-provoking and caused users to withdraw [48]. Similarly, loss of members within a lung cancer OSG evoked grief, distress, and concern for the future [145]. At the same time, patients with metastatic breast cancer felt silenced, marginalized, and helpless in mixed-stage OSGs, whereas in stage-specific OSGs, they were able to talk openly and felt understood [53]. While group cohesion can develop across diagnostically dissimilar professionally moderated support groups [219], these findings suggest that stage-specific groups might be more effective for unmoderated OSGs.

Importantly, our findings indicate that the nature and content of posts in OSGs may have the greatest effect on outcomes. Several studies of cancer-specific and mixed-cancer OSGs concluded that the most beneficial posts were those that involved the sharing of feelings and experiences [49,50,54,56,57,60,66,99,101,106,107,119,121,122,126-128,150,153,154,156,167,172,178-181,184,189,191,222]. Emotionally expressive writing is associated with positive psychosocial adjustment and well-being [223], and such disclosures foster feelings of connection and group cohesion [224]. However, expressing emotions is not enough to benefit maximally from an OSG. Several studies of breast cancer OSGs found that posts that express emotions and cognitive processing confer the most benefit for the poster and reader [150,184,189,191]. For example, posts in breast cancer OSGs that included a higher percentage of insightful disclosure words (eg, aware, know, realize, think, and understand) led to greater positive effects that were maintained over time [184,191]. Additionally, insightful disclosure posts were more likely to generate empathetic responses in an OSG for young adults with cancer [182]. Hence, posts that include emotional disclosures and cognitive processing are most helpful for the poster and the reader, and most likely to garner a helpful empathetic response, leading to further benefits. These findings highlight the importance of crafting messages for maximum benefit. However, for the health benefits to manifest, OSGs must provide a safe environment for disclosing emotions and stigmatizing experiences [224]. Community rules can establish acceptable behavior and foster a safe online environment, while anonymity and strong privacy controls can facilitate disclosure.

The extent to which an individual may benefit from OSGs also depends on how they participate, which is influenced by technology factors, group process, and coping style. To be most effective, OSGs need to be active to ensure users can access support when needed, enhancing perceptions of control. Hence, larger groups may be more effective because there are more active members to post and respond. However, ideal group size depends on modality, as live chats can become overwhelming with too many participants and posts [100]. While anonymity contributes to activity in OSGs by facilitating disclosure, it can also contribute to inappropriate behaviors [225]. In this context, people feel less restrained and, without visual cues, do not see the discomfort they may cause others [225]. OSGs that are active may also be more trustworthy because members can participate in regulating the accuracy of the posts. For example, in a breast cancer OSG, only 10 of 4600 posts were found to be false or potentially misleading, and 7 of them were corrected by OSG members within 4 hours of posting [226].

Ongoing investment in moderator training and facilitation guidelines covering conflict resolution, information verification, and cultural sensitivity is recommended [88,144,152]. Some studies report that patients with cancer prefer professionally moderated OSGs for fear of misinformation [99,151]. However, studies comparing peer vs professionally led breast cancer OSGs have found no differences in outcomes [165,196]. Some studies also suggest that people who participate actively in OSGs by posting benefit more than those who participate less actively or passively by lurking [106,191]. However, other studies found that lurkers benefit to a similar degree [227]. Variations in coping styles may be responsible for the variable effects of OSG participation. For example, frequent users of a breast cancer OSG who engaged in emotionally expressive coping (eg, actively expressed and processed emotions) experienced better psychological well-being than frequent users who approached their emotions less actively [106]. Engaging the community in implementing community guidelines and offering users tips on how to participate for maximum benefit could optimize the effects of cancer OSGs.

### Who Is Most Likely to Benefit From OSGs and Why?

Overall, the evidence suggests that the people most likely to benefit from cancer OSGs are those who have unmet supportive care needs, perceive OSGs to be useful and easy to use, can find an OSG to which they feel connected, and that meets their needs. This means that people who have greater illness uncertainty (eg, newly diagnosed, experiencing new symptoms, or advancing disease), have a complex medical condition, greater symptom distress or social stigma associated with their condition, and lack sufficient support from offline family and friends, health care professionals, and other patients with cancer, may benefit more from cancer OSGs. Individuals with altruistic support needs, who are doing and coping well and who want to give back by supporting other patients with cancer in OSGs, can also benefit from OSGs.

In addition, most studies to date have examined cancer OSGs for women with breast cancer (71/92, 77% of single cancer studies) or have involved women with breast cancer (46/50, 92% of mixed cancer studies). This may mean that there are more OSGs available for women with breast cancer than for other people with other cancer types. Hence, women affected by breast cancer may have an easier path to benefitting from OSGs at present because there are likely more options for them to choose from to meet their needs.

The findings of this review also suggest that an individual’s coping style may impact whether they use and benefit from OSGs. Seeking information and support is reflective of an active coping style. Women are more likely to engage in active coping strategies and seek support than men [228], which may in part explain prior work demonstrating that women are more likely to use social media for cancer-related support than men [214]. Correspondingly, a population-based study of men with prostate cancer reported that active information seekers were 4.5 times more likely to use the internet as a source of information and support than those who were not [229].

Individuals with emotion-focused coping styles may also benefit more from OSGs, given that the most beneficial posts are those that express emotions and cognitive processing. However, studies of breast and prostate cancer OSGs have documented more information than emotionally supportive talk occurring in cancer OSGs [49,230]. Studies of breast, prostate, and mixed cancer OSGs have also identified gender differences, with some reporting that men are more likely to share information while women are more likely to share emotional support [131,166,230]. In contrast, the discourse analysis of ovarian and prostate cancer OSGs by Sullivan [49] concluded that both genders provide information and emotional support in OSGs, albeit in different ways. Men tended to engage in “report-talk,” providing information in the form of personal case histories, and demonstrated empathy by ensuring the information shared was accurate. In contrast, women more often engaged in “rapport-talk,” providing information and emotional support through the sharing of personal experiences and feelings.

However, not all patients with cancer seek support online or from OSGs specifically. Most studies included in this review reported that participants were college- or university-educated, age was variable, and income was inconsistently reported. Age, education, income, and broadband access remain well-known predictors of internet use [231]. In prior work involving patients with breast, testicular, and head and neck cancer in Canada, 5 variables correlated with OSG use: age, first spoken language, education, treatments received, and unmet supportive care needs [232-234]. In another study, confidence using the internet was associated with use of social media as a resource for cancer information and support [214]. This review similarly found that lower socioeconomic status may limit some individuals’ access to OSGs due to a lack of stable housing, internet access, or eHealth literacy.

Lastly, it is unclear how and to what extent patients with cancer and survivors of cancer who identify with non-White racial and ethnic backgrounds benefit from OSGs. Most studies (101/168, 60.1%) did not specify the racial or ethnic background of study participants. Of those that did, most studies (51/67, 76%) reported that most study participants were White North American or European. These findings mirror those of a review of cancer OSGs conducted over 20 years ago [140], indicative of little progress toward understanding the use and effects of English-language cancer OSGs among racialized ethnic groups and different linguistic communities. There was also a lack of studies on the use and effects of OSGs among 2SLGBTQIA+ (2-spirit, lesbian, gay, bisexual, transgender, queer, intersex, or asexual) patients with cancer. Only 2 such studies were identified: 1 focused on the OSG experiences of sexual minority women with breast cancer [159], and the other focused more broadly on the OSG experiences of sexual minority women with cancer [235]. Both highlighted the importance of designing OSGs to promote inclusivity and acceptance of diverse sexual and gender identities.

### Implications and Recommendations

This study confirms and extends the multitheory framework we initially proposed to explain the use and effects of breast cancer OSGs. In doing so, the findings demonstrate the value of social support and technology adoption theory in explaining how online support interventions influence health, and realist methodology in uncovering the mechanisms through which complex social interventions exert their effects. Further research, as detailed below, is needed to test the proposed program theory.

The review identified several gaps in the literature. Most of the literature to date has focused on the use and effects of cancer OSGs among White, cisgender, heterosexual women with breast cancer from North America or Europe. Further research is needed to better understand and explore the applicability of our findings among men, ethnically and linguistically diverse minorities and majorities, 2SLGBTQIA+ patients with cancer, and people diagnosed with other common cancers, rarer cancers, and niche cancer groups at different stages of the cancer continuum. In addition, as most studies (109/168, 64.9%) were performed in the United States, further research is needed to determine how OSGs work for people in non-English speaking countries.

There is also a need for more research on the nature of posts that confer the most benefit for diverse gender and ethnocultural groups, how OSGs can be configured and moderated to optimize supportive exchanges for maximal benefit, and interventions designed to maximize benefits. The quality of reporting on the moderation of OSGs is generally poor, leaving many unanswered questions with respect to the training and qualifications of moderators and the extent and style of moderation.

Lastly, further research is needed to explore the implications of new technologies such as virtual reality (VR) and artificial intelligence (AI) in OSGs. VR could remove physical barriers to support, enabling “face-to-face” interactions through avatars and offering an interactive experience, but despite its potential, VR adoption may be limited due to low familiarity and accessibility [144]. In a series of 3 studies, Leung et al [236-238] demonstrated the potential of an AI cofacilitator to recommend resources, detect distress, and facilitate group cohesion in synchronous cancer OSGs with promising findings. The use of AI to support online content moderation raises ethical concerns regarding privacy, free speech, and bias [239]. Guidelines are needed for the use of AI in OSGs to balance the potential benefits with ethical considerations to ensure safe and inclusive online environments.

### Strengths and Limitations

We followed a rigorous and systematic procedure to synthesize and analyze a substantial body of research using best practices in realist reviews, with guidance from an expert in realist methodologies and 5 categories of knowledge users. The findings consolidate and confirm prior research and provide new insight into the mechanisms underlying the effectiveness of OSGs and practical implications for improvement. However, as with any review method, the resulting synthesis is only as good as the primary data on which it is built. We limited our search to studies published in English, which in most cases focused on English-language OSGs. Another limitation we encountered in our review was that most studies of OSGs focused on women with breast cancer and included a disproportionate number of White, well-educated, and above-average-income earners. The predominance of breast cancer-related data may limit the generalizability of findings; however, using a realist approach enabled us to draw on data across cancer types, which showed commonalities. Second, many studies did not describe the OSGs in sufficient detail, including the technology used, the characteristics of the participants, the training and qualifications of the moderator, and the moderation strategy, and did not follow the CONSORT-EHEALTH (Consolidated Standards of Reporting Trials of Electronic and Mobile Health Applications and Online Telehealth) reporting guidelines [240]. While some studies predate these reporting guidelines, many did not. To improve reporting of OSG interventions, we recommend updating the CONSORT-EHEALTH guidelines to capture the contextual aspects of social-media-based interventions such as OSGs that influence their effects.

### Conclusions

OSGs can be an effective source of support for patients with cancer and survivors of cancer that can address many supportive care needs and improve psychosocial well-being. However, outcomes depend on specific contexts and mechanisms that impact how well OSGs meet the needs of users. To maximize the effectiveness of OSGs, we recommend optimizing the design and implementation of OSGs to (1) help assess fit and address specific needs; (2) demonstrate trustworthiness; (3) enhance anonymity and control, and protect privacy; (4) enhance ease-of-use; (5) support connection and belonging; (6) encourage activity; (7) enhance the nature of content shared to boost therapeutic effects; and (8) monitor and adjust design and management strategies to optimize effectiveness.

## Supplementary material

10.2196/77445 Multimedia Appendix 1 MEDLINE search strategy.

10.2196/77445 Multimedia Appendix 2 Study designs and demographics.

10.2196/77445 Multimedia Appendix 3 OSG characteristics. OSG: online support group.

10.2196/77445 Checklist 1 RAMESES checklist.

## Abbreviations

2SLGBTQIA+: 2-spirit, lesbian, gay, bisexual, transgender, queer, intersex, or asexual
AI: artificial intelligence
CMOC: context-mechanism-outcome configuration
CONSORT-EHEALTH: Consolidated Standards of Reporting Trials of Electronic and Mobile Health Applications and Online Telehealth
OSG: online support group
PRESS: Peer Review of Electronic Search Strategy
PROSPERO: International Prospective Register of Systematic Review
RAMESES: Realist and Meta-Narrative Evidence Syntheses: Evolving Standards
TAM: Technology Acceptance Model
VR: virtual reality
