# Supplementary material for: How Cancer Online Support Groups Work, for Whom, and in What Circumstances: Realist Review
Source: J Med Internet Res. 2026 May 13;28:e77445. doi: 10.2196/77445 (PMC13170744; doi:10.2196/77445)
Supplement: Multimedia Appendix 1 — MEDLINE search strategy. [file jmir-v28-e77445-s001.docx]

**MEDLINE Search Strategy (Ovid Platform)**

| # | Searches |
| --- | --- |
| 1 | Telemedicine/ |
| 2 | (telemedicine or tele-medicine).ti,ab,kw. |
| 3 | exp Computer Communication Networks/ |
| 4 | exp Internet/ |
| 5 | internet access/ [ MeSH 2019 ] |
| 6 | Internet-based intervention/ [ MeSH 2020 ] |
| 7 | "Internet Use"/ [ MeSH 2021 ] |
| 8 | "Internet of Things"/ [ MeSH 2020 ] |
| 9 | exp Electronic Mail/ |
| 10 | Mobile Applications/ |
| 11 | Online systems/ |
| 12 | Smartphone/ |
| 13 | "Cell Phone Use"/ |
| 14 | Cell Phones/ |
| 15 | (information adj2 highway*).mp. |
| 16 | cyber*.mp. |
| 17 | electronic mail*.mp. |
| 18 | email*.mp. |
| 19 | e-mail*.mp. |
| 20 | internet*.mp. |
| 21 | web bas*.mp. |
| 22 | web page*.mp. |
| 23 | web site*.mp. |
| 24 | webbas*.mp. |
| 25 | webpage*.mp. |
| 26 | website*.mp. |
| 27 | world wide web*.mp. |
| 28 | www.mp. |
| 29 | Blogging/ |
| 30 | Social Media/ |
| 31 | Online Social Networking/ [ MeSH 2019 ] |
| 32 | Patient Portals/ |
| 33 | Semantic Web/ |
| 34 | "e-patient*".mp. |
| 35 | "health 2.0".mp. |
| 36 | "internet of things".mp. |
| 37 | "ning.com".mp. |
| 38 | "second life".mp. |
| 39 | "web 2.0".mp. |
| 40 | "www.ning.com".mp. |
| 41 | "www.patientslikeme.com".mp. |
| 42 | (android adj2 (app or apps or application?)).mp. |
| 43 | (apple adj2 (app or apps or application?)).mp. |
| 44 | (communicat* adj10 platform?).mp. |
| 45 | (digital* adj10 platform?).mp. |
| 46 | (digital?? adj6 outlet?).mp. |
| 47 | (digital?? adj6 portal?).mp. |
| 48 | (electronic adj2 group*).mp. |
| 49 | (health care app or health care apps).mp. |
| 50 | (health* app or health* apps).mp. |
| 51 | (internet adj2 group*).mp. |
| 52 | (internet adj2 support*).mp. |
| 53 | (online adj2 account???).mp. |
| 54 | (on-line adj2 account???).mp. |
| 55 | (online adj2 group*).mp. |
| 56 | (on-line adj2 group*).mp. |
| 57 | (online adj2 support*).mp. |
| 58 | (on-line adj2 support*).mp. |
| 59 | (portable adj2 (app or apps or application?)).mp. |
| 60 | (Sina adj2 blog*).mp. |
| 61 | (Sina adj2 microblog*).mp. |
| 62 | (smart adj2 (phone? or device? or app or apps or application?)).mp. |
| 63 | (sociable adj2 technolog*).mp. |
| 64 | (social adj2 site?).mp. |
| 65 | (social adj2 software*).mp. |
| 66 | (social adj2 technolog*).mp. |
| 67 | (social adj2 utilit*).mp. |
| 68 | (social adj2 website?).mp. |
| 69 | (social* adj10 platform?).mp. |
| 70 | (virtual adj2 collabor*).mp. |
| 71 | (virtual adj2 communit*).mp. |
| 72 | (virtual adj2 group*).mp. |
| 73 | (virtual adj2 support*).mp. |
| 74 | (web adj2 app?).mp. |
| 75 | (web adj2 application?).mp. |
| 76 | (web adj2 group*).mp. |
| 77 | (web adj2 support*).mp. |
| 78 | (web* adj2 blog*4).mp. |
| 79 | (web? adj2 log*3).mp. |
| 80 | (webbased adj2 application?).mp. |
| 81 | (web-based adj2 application?).mp. |
| 82 | (website* adj2 blog*4).mp. |
| 83 | (yahoo* adj2 group?).mp. |
| 84 | bebo.mp. |
| 85 | blog*.mp. |
| 86 | blogspot?.mp. |
| 87 | bulletin board system*.mp. |
| 88 | chat group*.mp. |
| 89 | chat room*.mp. |
| 90 | chat technolog*.mp. |
| 91 | chatgroup*.mp. |
| 92 | chatroom*.mp. |
| 93 | digital tablet?.mp. |
| 94 | discussion list*.mp. |
| 95 | ebulletin board*.mp. |
| 96 | e-bulletin board*.mp. |
| 97 | ecommunit*.mp. |
| 98 | e-communit*.mp. |
| 99 | electronic group*.mp. |
| 100 | electronic support*.mp. |
| 101 | electronic tablet?.mp. |
| 102 | epatient*.mp. |
| 103 | FaceBook*.mp. |
| 104 | FaceTime??.mp. |
| 105 | Flickr.mp. |
| 106 | google android.mp. |
| 107 | Google Group?.mp. |
| 108 | Google Hangout?.mp. |
| 109 | google pixel?.mp. |
| 110 | google play.mp. |
| 111 | Google Plus.mp. |
| 112 | Google Wave.mp. |
| 113 | GooglePlus.mp. |
| 114 | health* application?.mp. |
| 115 | health* tech* enable?.mp. |
| 116 | Instagram??.mp. |
| 117 | instant messag*.mp. |
| 118 | inter* relay*.mp. |
| 119 | internet group*.mp. |
| 120 | internet* tablet?.mp. |
| 121 | (irc and relay).mp. |
| 122 | Kaixin001.mp. |
| 123 | list serv*.mp. |
| 124 | listserv*.mp. |
| 125 | livejournal.mp. |
| 126 | mail box*.mp. |
| 127 | mail??? list*.mp. |
| 128 | mailbox*.mp. |
| 129 | mashup*.mp. |
| 130 | meebo.mp. |
| 131 | message board*.mp. |
| 132 | Messageboard*.mp. |
| 133 | microblog*.mp. |
| 134 | micro-blog*.mp. |
| 135 | Microsoft Surface??.mp. |
| 136 | mixi.mp. |
| 137 | myspace*.mp. |
| 138 | news group*.mp. |
| 139 | newsgroup*.mp. |
| 140 | ning.mp. |
| 141 | orkut.mp. |
| 142 | patientslikeme*.mp. |
| 143 | personali#ed health*.mp. |
| 144 | phone app?.mp. |
| 145 | phone application?.mp. |
| 146 | Pinterest??.mp. |
| 147 | Quora.mp. |
| 148 | Reddit??.mp. |
| 149 | secondlife.mp. |
| 150 | semantic web?.mp. |
| 151 | Sina Weibo.mp. |
| 152 | SinaWeibo??.mp. |
| 153 | Skype?.mp. |
| 154 | smartphon*.mp. |
| 155 | SnapChat??.mp. |
| 156 | social awareness*.mp. |
| 157 | tablet? computer?.mp. |
| 158 | Taringa?.mp. |
| 159 | Tumblr.mp. |
| 160 | tweet.mp. |
| 161 | twitter.mp. |
| 162 | usenet*.mp. |
| 163 | virtual communit*.mp. |
| 164 | virtual support*.mp. |
| 165 | webcam*.mp. |
| 166 | web-cam*.mp. |
| 167 | weblog*.mp. |
| 168 | web-log*.mp. |
| 169 | WeChat??.mp. |
| 170 | WhatsApp??.mp. |
| 171 | wiki*.mp. |
| 172 | wordpress*.mp. |
| 173 | youtube*.mp. |
| 174 | Zoom??.mp. |
| 175 | or/1-174 [ Internet / Internet Support Sites and related terms ] |
| 176 | exp Social Networking/ |
| 177 | exp Self-Help Groups/ |
| 178 | Patient Participation/ |
| 179 | Patient Reported Outcome Measures/ |
| 180 | Peer Group/ |
| 181 | Peer Influence/ |
| 182 | Self-help Groups/ |
| 183 | Social Support/ |
| 184 | Survivors/ |
| 185 | (patient?? adj2 communit*).mp. |
| 186 | (patient?? adj2 outcome?).mp. |
| 187 | (peer?? adj4 influenc*).mp. |
| 188 | (social adj2 network*4).tw. |
| 189 | discuss* group*.mp. |
| 190 | (patient? and (fora or forum or forums)).mp. |
| 191 | (group?? adj2 participa*).mp. |
| 192 | (group? adj2 patient?).mp. |
| 193 | p2p.mp. |
| 194 | ((patient? or family or families or carer? or caregiver? or care giver? or survivor?) and (peer or peers)).mp. |
| 195 | (peer? adj2 group*).mp. |
| 196 | (peer? adj2 relation*).mp. |
| 197 | peer support*.mp. |
| 198 | peer to peer*.mp. |
| 199 | selfhelp*.mp. |
| 200 | self-help*.mp. |
| 201 | social support*.mp. |
| 202 | support group*.mp. |
| 203 | survivor*.mp. |
| 204 | or/176-203 [ Peer/Health Communities ] |
| 205 | (communit* adj2 (internet* or world wide web* or e-mail* or email* or electronic mail* or web pag* or webpage* or web site* or website* or web base* or www or cyber* or online or on-line)).mp. |
| 206 | 175 and (204 or 205) [ Internet/Internet Support + Peer/Health Communities ] |
| 207 | Cancer Survivors/ |
| 208 | ((cancer* or carcino* or neoplas* or onco*) and (survivor or survivors or survivorship*)).mp. |
| 209 | exp neoplasms/ or American Cancer Society/ or exp angiogenesis inducing agents/ or exp antibodies, neoplasm/ or exp antigens, neoplasm/ |
| 210 | exp antineoplastic agents/ or exp antineoplastic protocols/ or exp biopsy/ or biopsy.tw,kw. or exp bone marrow purging/ or exp bone marrow transplantation/ or exp cancer care facilities/ or exp cancer vaccines/ or exp carcinogenicity tests/ or exp carcinogens/ |
| 211 | exp chemoembolization, therapeutic/ or exp colonography, computed tomographic/ or exp colonoscopy/ or exp colposcopy/ or exp combined modality therapy/ or exp cryosurgery/ or exp cytapheresis/ or exp dna, neoplasm/ or exp drug resistance, neoplasm/ or exp drug screening assays, antitumor/ or exp early detection of cancer/ or exp gene expression regulation, neoplastic/ or exp genes, neoplasm/ |
| 212 | exp graft vs tumor effect/ or exp hematopoietic stem cell transplantation/ or exp hematopoietic stem cell mobilization/ or exp immunotherapy, adoptive/ or exp leukostasis/ or exp lymph node excision/ or exp lymphocytes, tumor-infiltrating/ or exp mammography/ or exp mastectomy/ or exp medical oncology/ or exp mohs surgery/ or exp myelodysplastic-myeloproliferative diseases/ |
| 213 | exp neoplasm proteins/ or exp neoplasm staging/ or exp neoplasm transplantation/ or exp neoplastic processes/ or exp neoplastic stem cells/ or exp oncogene fusion/ or exp oncogenic viruses/ or exp oncologic nursing/ or exp oncology service, hospital/ or exp oncolytic viruses/ or exp papillomavirus vaccines/ |
| 214 | exp peripheral blood stem cell transplantation/ or exp radiotherapy/ or exp radiotherapy planning, computer assisted/ or exp rna, neoplasm/ or exp second-look surgery/ or exp SEER program/ or stem cell transplantation/ or exp transplantation conditioning/ or exp tumor cells, cultured/ or exp tumor escape/ or exp tumor lysis syndrome/ |
| 215 | exp tumor markers, biological/ or exp tumor necrosis factors/ or exp receptors, tumor necrosis factor/ |
| 216 | exp "Tumor Necrosis Factor Receptor-Associated Peptides and Proteins"/ |
| 217 | exp ultrasonography, mammary/ |
| 218 | (AACR or AJCC or (ASCO not fungi) or IARC or "National Cancer Institute" or NCI or UICC or aCML or AGCUS or AILD or AML or ANLL or ASCUS or ATLL or BRCA or BRCA1 or BRCA2 or CIN or CLL or CMML or CMPD or ECCL or EGIST or FMTC or GLNH or HNPCC or HNSCC or HPV or HSIL or ICD O or JCML or JMML or LGLL or MGUS or MLH1 or MPD or MSH2 or NSCLC or RAEB or RCMD).tw,kw. |
| 219 | (SCLC or VOD or 5q syndrome or BCR ABL or c erbB 2 or c erbB2 or carney complex or cone biopsy or denys drash or essential thrombocythemia or estrogen receptor negative or estrogen receptor positive or li fraumeni or meigs syndrome or molar pregnancy or mycosis fungoides or peutz jeghers or sentinel lymph node or sezary syndrome or struma ovarii or sturge weber or zollinger ellison).tw,kw. |
| 220 | ((aberrant and crypt and foci) or (barrett and esophagus) or (gestational and trophoblastic) or (microsatellite and instability) or (paget and (breast or nipple)) or (polycythemia and vera) or (radiation and therapy) or (WAGR and syndrome) or (pap and (smear or smears)) or (Papanicolaou or cervical smear or cervical smears or pap test or pap tests) or (PSA and prostate) or PSA test or PSA testing or (prostate and specific and antigen)).tw,kw. |
| 221 | (acanthoma or acanthomas or acrochordon or acrochordons or acrospiroma or acrospiromas or adamantinoma or adamantinomas or adenoacanthoma or adenoacanthomas or adenoameloblastoma or adenoameloblastomas or adenocanthoma or adenocanthomas).tw,kw. |
| 222 | (adenocarcinoma or adenocarcinomas or adenofibroma or adenofibromas or adenolipoma or adenolipomas or adenolymphoma or adenolymphomas or adenoma or adenomas or adenomatosis or adenomatous or adenomyoepithelioma or adenomyoepitheliomas or adenomyoma or adenomyomas or adenosarcoma or adenosarcomas or adenosis).tw,kw. |
| 223 | (aesthesioneuroblastoma or aesthesioneuroblastomas or ameloblastoma or ameloblastomas or amyloidoses or amyloidosis or androblastoma or androblastomas or angioblastoma or angioblastomas or angioendothelioma or angioendotheliomas or angioendotheliomatosis or angiofibroma or angiofibromas or angiofibrosarcoma or angiogenesis factor or angiokeratoma or angiokeratomas or angioleiomyoma or angioleiomyomas or angiolipoma or angiolipomas or angioma or angiomas).tw,kw. |
| 224 | (angiolipoma or angiolipomas or angioma or angiomas or angiomatosis or angiomyolipoma or angiomyolipomas or angiomyoma or angiomyomas or angiomyxoma or angiomyxomas or angioreticuloma or angioreticulomas or angiosarcoma or angiosarcomas or anticancer or anticarcinogenesis or anticarcinogenic or antimutagenesis or antineoplastic).tw,kw. |
| 225 | (antioncogene or antioncogenes or antitumor or antitumors or antitumour or antitumours or apudoma or apudomas or argentaffinoma or argentaffinomas or arrhenoblastoma or arrhenoblastomas or astroblastoma or astroblastomas or astrocytoma or astrocytomas or astroglioma or astrogliomas or atypia).tw,kw. |
| 226 | (baltoma or basiloma or basilomas or biochemotherapies or biochemotherapy or bioradiotherapy or Birt-Hogg-Dube or blastoma or blastomas or cachexia or cancer or cancerous or cancers or carcinogen or carcinogenesis or carcinogenic or carcinogens or carcinoid or carcinoma or carcinomas or carcinomatosis or carcinosarcoma or carcinosarcomas).tw,kw. |
| 227 | (cavernoma or cavernomas or cementoma or cementomas or cerbB2 or ceruminoma or ceruminomas or chemodectoma or chemodectomas or chemoimmunoradiotherapy or chemoimmunotherapies or chemoimmunotherapy or chemoprevention or chemoradiation or chemoradiotherapies or chemoradiotherapy or cherubism or chloroma or chloromas or cholangiocarcinoma).tw,kw. |
| 228 | (cholangiocarcinomas or cholangiohepatoma or cholangioma or cholangiomas or cholangiosarcoma or cholesteatoma or cholesteatomas or chondroblastoma or chondroblastomas or chondroma or chondromas or chondrosarcoma or chondrosarcomas or chordoma or chordomas or chorioadenoma or chorioadenomas or chorioangioma or chorioangiomas or choriocarcinoma).tw,kw. |
| 229 | (choriocarcinomas or chorioepithelioma or chorioepitheliomas or chorionepithelioma or chorionepitheliomas or choristoma or choristomas or chromaffinoma or chromaffinomas or collagenoma or collagenomas or colonoscopies or coloscopy or coloscopies or comedocarcinoma or comedocarcinomas or condyloma or condylomas or corticotropinoma or corticotropinomas).tw,kw. |
| 230 | (craniopharyngioma or craniopharyngiomas or cylindroma or cylindromas or cyst or cysts or cystadenocarcinoma or cystadenocarcinomas or cystadenofibroma or cystadenofibromas or cystadenoma or cystadenomas or cystoma or cystomas or cystosarcoma or cystosarcomas or cysts).tw,kw. |
| 231 | (dentinoma or dentinomas or dermatofibroma or dermatofibromas or dermatofibrosarcoma or dermatofibrosarcomas or dermoid or desmoid or desmoplastic or dictyoma or dysgerminoma or dysgerminomas or dyskeratoma or dyskeratomas or dysmyelopoiesis or dysplasia or dysplastic).tw,kw. |
| 232 | (ectomesenchymoma or ectomesenchymomas or elastofibroma or elastofibromas or enchondroma or enchondromas or enchondromatosis or endothelioma or endotheliomas or ependymoblastoma or ependymoblastomas or ependymoma or ependymomas or epidermoid or epithelioma or epitheliomas).tw,kw. |
| 233 | (erythroleukaemia or erythroleukaemias or erythroleukemia or erythroleukemias or erythroplakia or erythroplakias or erythroplasia or esthesioneuroblastoma or esthesioneuroblastomas or esthesioneuroepithelioma or esthesioneuroepitheliomas or exostosis or fibroadenoma or fibroadenomas).tw,kw. |
| 234 | (fibroadenosarcoma or fibroadenosis or fibrochondrosarcoma or fibroelastoma or fibroelastomas or fibroepithelioma or fibroepitheliomas or fibrofolliculoma or fibrofolliculomas or fibroid or fibroids or fibrolipoma or fibrolipomas).tw,kw. |
| 235 | (fibroliposarcoma or fibroma or fibromas or fibromatosis or fibromyoma or fibromyomas or fibromyxolipoma or fibromyxoma or fibromyxomas or fibroodontoma or fibroodontomas or fibrosarcoma or fibrosarcomas or fibrothecoma or fibrothecomas or fibroxanthoma or fibroxanthomas or fibroxanthosarcoma or fibroxanthosarcomas).tw,kw. |
| 236 | (ganglioblastoma or ganglioblastomas or gangliocytoma or gangliocytomas or ganglioglioma or gangliogliomas or ganglioneuroblastoma or ganglioneuroblastomas or ganglioneurofibroma or ganglioneurofibromas or ganglioneuroma or ganglioneuromas or gastrinoma or gastrinomas or germinoma or germinomas or glioblastoma or glioblastomas or gliofibroma or gliofibromas or glioma or gliomas or gliomatosis).tw,kw. |
| 237 | (glioneuroma or glioneuromas or gliosarcoma or gliosarcomas or glomangioma or glomangiomas or glomangiomatosis or glomangiomyoma or glomangiomyomas or glomangiosarcoma or glomangiosarcomas or glucagonoma or glucagonomas or gonadoblastoma or gonadoblastomas or gonocytoma or gonocytomas or granuloma or granulomas or granulomatosis or gynaecomastia or gynandroblastoma or gynecomastia).tw,kw. |
| 238 | (haemangioblastoma or haemangioblastomas or haemangioma or haemangiomas or haemangiopericytoma or haemangiopericytomas or haemangiosarcoma or haemangiosarcomas or hamartoma or hamartomas or hemangioblastoma or hemangioblastomas or hemangioendothelioma or hemangioendotheliomas or hemangioendotheliosarcoma or hemangioendotheliosarcomas or hemangioma or hemangiomas or hemangiomatosis).tw,kw. |
| 239 | (hemangiopericytoma or hemangiopericytomas or hemangioperithelioma or hemangiosarcoma or hemangiosarcomas or hepatoblastoma or hepatoblastomas or hepatocarcinoma or hepatocarcinomas or hepatocholangiocarcinoma or hepatocholangiocarcinomas or hepatoma or hepatomas or hibernoma or hibernomas or hidradenoma).tw,kw. |
| 240 | (hidradenomas or hidrocystoma or hidrocystomas or histiocytoma or histiocytomas or hodgkin or hodgkins or hydatidiform or hydradenoma or hydradenomas or hypernephroma or hypernephromas).tw,kw. |
| 241 | (immunochemoradiotherapy or immunochemotherapies or immunochemotherapy or immunocytoma or immunocytoma or immunoradiotherapy or insulinomas or keratoacanthoma or keratoacanthomas or keratosis or leiomyoblastoma or leiomyoblastomas or leiomyofibroma or leiomyofibromas or leiomyoma or leiomyomas or leiomyomatosis or leiomyosarcoma or leiomyosarcomas or leukaemia or leukaemias or leukemia or leukemias or leukoplakia or leukoplakias or lipoadenoma or lipoadenomas or lipoblastoma or lipoblastomas).tw,kw. |
| 242 | (lipoblastomatosis or lipoma or lipomas or lipomatosis or liposarcoma or liposarcomas or luteinoma or luteoma or luteomas or lymphangioendothelioma or lymphangioendotheliomas).tw,kw. |
| 243 | (lymphangioleiomyomatosis or lymphangioma or lymphangiomas or lymphangiomatosis or lymphangiomyoma or lymphangiomyomas or lymphangiomyomatosis or lymphangiosarcoma or lymphangiosarcomas or lymphoepithelioma or lymphoepitheliomas or lymphoma or lymphomas or lymphoproliferation or lymphoproliferations or lymphoproliferative or lymphoscintigraphic or lymphoscintigraphy).tw,kw. |
| 244 | (macroglobulinemia or macroglobulinemias or macroprolactinoma or malignancies or malignancy or malignant or maltoma or maltomas or mammogram or mammograms or masculinovoblastoma or mastocytoma or mastocytomas or mastocytosis or medulloblastoma or medulloblastomas or medullocytoma or medullocytomas or medulloepithelioma or medulloepitheliomas or medullomyoblastoma or medullomyoblastomas or melanoacanthoma).tw,kw. |
| 245 | (melanoacanthomas or melanoameloblastoma or melanocytoma or melanocytomas or melanoma or melanomas or melanomatosis or meningioblastoma or meningioma or meningiomas or meningiomatosis or mesenchymoma or mesenchymomas or mesonephroma or mesonephromas or mesothelioma or mesotheliomas or metaplasia or metastases or metastasis or metastatic or microglioma or microgliomas or micrometastases).tw,kw. |
| 246 | (micrometastasis or mucositis or myelodysplasia or myelodysplasias or myelodysplastic or myelofibrosis or myelolipoma or myelolipomas or myeloma or myelomas or myelomatosis or myeloproliferation or myeloproliferations or myeloproliferative or myelosuppression or myoblastoma or myoblastomas or myoepithelioma or myoepitheliomas or myofibroblastoma or myofibroblastomas or myofibroma).tw,kw. |
| 247 | (myofibromas or myofibromatosis or myofibrosarcoma or myofibrosarcomas or myolipoma or myolipomas or myoma or myomas or myosarcoma or myosarcomas or myxofibroma or myxofibromas or myxolipoma or myxolipomas or myxoliposarcoma or myxoma or myxomas or naevus or neoplasia or neoplasia or neoplasm or neoplasms or neoplastic or nephroblastoma or nephroblastomas or neurilemmoma or neurilemmomas).tw,kw. |
| 248 | (neurilemmomatosis or neurilemoma or neurilemomas or neurinoma or neurinomas or neuroblastoma or neuroblastomas or neurocytoma or neurocytomas or neuroepithelioma or neuroepitheliomas or neurofibroma or neurofibromas or neurofibromatosis or neurofibrosarcoma or neurofibrosarcomas or neurolipocytoma or neuroma or neuromas or neuronevus or neurothekeoma or neurothekeomas or nevus or nonhodgkin or nonhodgkins or nonseminoma or nonseminomas or nonseminomatous).tw,kw. |
| 249 | (odontoameloblastoma or odontoma or oligoastrocytoma or oligoastrocytomas or oligodendroglioma or oligodendrogliomas or oncocytoma or oncocytomas or oncogen or oncogene or oncogenes or oncogenesis or oncogenic or oncogens or oncologic or oncologist or oncologists or oncology or oncoprotein or oncoproteins or opsoclonus-myoclonus).tw,kw. |
| 250 | (orchioblastoma or orchioblastomas or osteoblastoma or osteoblastomas or osteochondroma or osteochondromas or osteochondrosarcoma or osteochondrosarcomas or osteoclastoma or osteoclastomas or osteofibrosarcoma or osteoma or osteomas or osteosarcoma or osteosarcomas).tw,kw. |
| 251 | (pancreatoblastoma or pancreatoblastomas or papilloma or papillomas or papillomata or papillomatosis or papillomavirus or papillomaviruses or parachordoma or parachordomas or paraganglioma or paragangliomas or paraneoplastic).tw,kw. |
| 252 | (perineurioma or perineuriomas or phaeochromocytoma or phaeochromocytomas or pheochromoblastoma or pheochromoblastomas or pheochromocytoma or pheochromocytomas or pilomatricoma or pilomatricomas or pilomatrixoma or pilomatrixomas or pinealblastoma or pinealoblastoma or pinealoblastomas or pinealoma or pinealomas or pineoblastoma or pineoblastomas or pineocytoma or pineocytomas or plasmacytoma or plasmacytomas or pneumoblastoma or pneumoblastomas or pneumocytoma).tw,kw. |
| 253 | (polyembryoma or polyembryomas or polyhistioma or polyhistiomas or polyp or polyposis or polyps or porocarcinoma or porocarcinomas or poroma or poromas or precancer or precancerous or preleukaemia or preleukaemias or preleukemia or preleukemias or premalignant or preneoplastic or prolactinoma or prolactinomas or protooncogene or protooncogenes or pseudotumor or pseudotumors).tw,kw. |
| 254 | (radiochemotherapy or radioimmunotherapies or radioimmunotherapy or reninoma or reninomas or reticuloendothelioma or reticuloendotheliomas or reticulohistiocytoma or reticulohistiocytomas or reticulosis or retinoblastoma or retinoblastomas or rhabdomyoma or rhabdomyomas or rhabdomyosarcoma or rhabdomyosarcomas or rhabdosarcoma or rhabdosarcomas or sarcoma or sarcomas or sarcomatosis).tw,kw. |
| 255 | (schwannoma or schwannomas or schwannomatosis or seminoma or seminomas or seminomatous or somatostatinoma or somatostatinomas or somatotropinoma or somatotropinomas or spermatocytoma or spiradenoma or spiradenomas or spongioblastoma or spongioblastomas or steatocystoma or steatocystomas or subependymoma or subependymomas or syringadenoma or syringadenomas or syringocystadenoma or syringocystadenomas or syringoma or syringomas).tw,kw. |
| 256 | (teratocarcinoma or teratocarcinomas or teratoma or teratomas or thecoma or thecomas or thymolipoma or thymolipomas or thymoma or thymomas or trichilemmoma or trichilemmomas or trichoadenoma or trichoblastoma or trichoblastomas or trichodiscoma or trichodiscomas or trichoepithelioma or trichoepitheliomas or trichofolliculoma or trichofolliculomas or tricholemmoma or tricholemmomas or tumor or tumorgenesis).tw,kw. |
| 257 | (tumorgenic or tumorigenesis or tumorigenic or tumorogenesis or tumorogenic or tumors or tumour or tumours or vipoma or vipomas or waldenstrom or waldenstroms or xanthoastrocytoma or xanthoastrocytomas or xanthofibroma or xanthofibromas or xanthogranuloma or xanthogranulomas or xanthoma or xanthomas or xanthosarcoma or xanthosarcomas).tw,kw. |
| 258 | or/207-257 [ ~~ Oncology hedge based on NLM/PubMed Cancer search ~~ ] |
| 259 | 206 and 258 [ Internet/Internet Support + Peer/Health Communities + Cancer ] |
| 260 | limit 259 to english language |
| 261 | (animal or animals or ape or apes or baboon or baboons or bat or bats or bird or birds or boar or boars or bonobo or bonobos or bovine or camel or camels or canine or canines or cat or cats or cattle or chicken or chickens or chimpanzee or chimpanzees or dog or dogs or dromedary or dromedaries or duck or ducks or equine or equines or feline or felines or ferret or ferrets or frog or frogs or fowl or fowls or goat or goats or hare or hares or hen or hens or horse or horses or lamb or lambs or livestock or macaque or macaques or mandrill or mandrills or mice or mink or minks or monkey or monkeys or mouse or murine or pig or pigs or piglet or piglets or poultry or porcine or orangutan or orangutans or rabbit or rabbits or rat or rats or rodent or rodents or sheep or swine or tamarin or tamarins or tiger or tigers or veterinary or veterinarian or veterinarians or waterfowl or waterfowls or weasel or weasels or veterinar* or (veterinar* or fish or shellfish)).ti,jw. |
| 262 | 260 not 261 |
| 263 | exp animals/ not (exp animals/ and exp humans/) |
| 264 | 260 not 263 |
| 265 | limit 260 to humans |
| 266 | 264 or 265 [ limiting to humans ] |
| 267 | 266 not ((monograph or textbook or study guide or preprint).pt. or (arxiv or medrxiv or biorxiv or research square).so. or (AHRQ* or StatPearls or Genereviewsovidsup).bt. or chapter.pr. or nb*.bk.) |
| 268 | remove duplicates from 267 [ removal of internal database duplicate citations ] |
| 269 | 268 [ Internet/Internet Support + Peer/Health Communities + Cancer; limits applied ] |
