# Supplementary material for: How Cancer Online Support Groups Work, for Whom, and in What Circumstances: Realist Review
Source: J Med Internet Res. 2026 May 13;28:e77445. doi: 10.2196/77445 (PMC13170744; doi:10.2196/77445)
Supplement: Multimedia Appendix 3 — OSG characteristics. OSG: online support group. [file jmir-v28-e77445-s003.docx]

**OSG Characteristics**

| **Reference #. Author(s) (Year)** | **Technology/**  **Intervention Type** | **Synchronous or Asynchronous** | **Moderators Present** | **Detail of Moderation Description** | **Moderator Type** |
| --- | --- | --- | --- | --- | --- |
| 1. Abioye et al (2024) | Not specified | Not specified | Yes – in one or more included interventions or intervention groups | Low | Trained professional(s)/staff and trained peers |
| 2. Adikari et al (2020) | Bulletin/Forum | Asynchronous | Not described | Not described | Not described |
| 3. Ahn and Lee (2023) | Not specified | Not specified | Not described | Not described | Not described |
| 4. Algtewi et al (2017) | Not specified | Not specified | Yes | Not described | Not described |
| 5. Baker et al (2011) | Multi-component website | Asynchronous | Yes – in one or more included interventions or intervention groups | Not described | Not described |
| 6. Batenburg and Das (2014) | Bulletin/forum | Asynchronous | Not described | Not described | Not described |
| 7. Batenburg and Das (2014) | Bulletin/forum | Asynchronous | Not described | Not described | Not described |
| 8. Baxter (2018) | Social media/networking website | Synchronous | Not described | Not described | Not described |
| 9. Beatty and Scott (2013) | Not specified | Not specified | Not described | Not described | Not described |
| 10. Bender et al (2013) | Multiple OSG types included | Not specified | Not described | Not described | Not described |
| 11. Broom (2005) | Not specified | Not specified | Not described | Not described | Not described |
| 12. Broom (2005) | Not specified | Not specified | Not described | Not described | Not described |
| 13. Cabling et al (2018) | Bulletin/forum | Asynchronous | Not described | Not described | Not described |
| 14. Changrani et al (2008) | Multi-component website | Both | Yes | Low | Trained professional(s)/staff |
| 15. Chen (2015) | Multi-component website | Asynchronous | Yes | Low | Lay peer(s) |
| 16. Chiu and Hsieh (2012) | Blog | Asynchronous | Not described | Not described | Not described |
| 17. Crook and Love (2017) | Bulletin/forum | Asynchronous | Not described | Not described | Not described |
| 18. Crook et al (2016) | Bulletin/forum | Asynchronous | Not described | Not described | Not described |
| 19. Dickerson et al (2006) | Multiple OSG types included | Both | Not described | Not described | Not described |
| 20. Dickerson et al (2011) | Not specified | Not specified | Not described | Not described | Not described |
| 21. Dolce (2011) | Not specified | Not specified | Not described | Not described | Not described |
| 22. Donovan et al (2014) | Bulletin/forum | Asynchronous | Not described | Not described | Not described |
| 23. Donovan et al (2019) | Social media/networking website | Asynchronous | Yes | High | Lay peer(s) |
| 24. Durant et al (2012) | Bulletin/forum | Asynchronous | Not described | Not described | Not described |
| 25. Erfani et al (2016) | Social media/networking website | Both | Yes | Low | Trained professional(s)/staff |
| 26. Erfani et al (2017) | Social media/networking website | Both | Not described | Not described | Not described |
| 27. Eysenbach (2003) | General internet use | Both | Not described | Not described | Not described |
| 28. Falisi et al (2017) | Multiple OSG types included | Both | Not described | Not described | Not described |
| 29. Fallon et al (2018) | Multicomponent website | Asynchronous | Yes | High | Trained professional(s)/staff |
| 30. Feng et al (2024) | Bulletin/forum | Asynchronous | Not described | Not described | Not described |
| 31. Fogel et al (2009) | Not specified | Not specified | Not described | Not described | Not described |
| 32. Foster and Roffe (2009) | Bulletin/forum | Asynchronous | Not described | Not described | Not described |
| 33. Frost et al (2014) | Multicomponent website | Asynchronous | Not described | Not described | Not described |
| 34. Garg et al (2020) | Bulletin/forum | Asynchronous | Not described | Not described | Not described |
| 35.Gill and Whisnant (2012) | Bulletin/forum | Asynchronous | Not described | Not described | Not described |
| 36. Ginossar (2008) | Mailing list | Asynchronous | Not described | Not described | Not described |
| 37. Gits et al (2013) | Multicomponent website | Asynchronous | Yes | High | Trained peer(s) |
| 38. Gooden and Winefield (2007) | Bulletin/forum | Asynchronous | Not described | Not described | Not described |
| 39. Gorlick et al (2014) | Multicomponent website | Both | Yes | High | Healthcare professional(s) |
| 40. Griffiths et al (2009) | Multiple OSG types included | Both | Yes – in one or more included interventions or intervention groups | Low | Healthcare professional(s) in one or more included interventions or intervention groups |
| 41. Griffiths et al (2015) | Multicomponent website | Asynchronous | Yes – in one or more included interventions or intervention groups | High | Not described |
| 42. Gupta and Schapira (2018) | Multiple OSG types included | Not specified | Yes – in one or more included interventions or intervention groups | Low | Healthcare/trained professional(s) in one or more included interventions or intervention groups |
| 43. Han et al (2012) | Multiple OSG types included | Asynchronous | Yes | Low | Trained professional(s)/staff |
| 44. Han et al (2014) | Multiple OSG types included | Asynchronous | Yes | High | Trained professional(s)/staff |
| 45. Han et al (2019) | Multiple OSG types included | Asynchronous | Not described | Not described | Not described |
| 46. Han et al (2011) | Multiple OSG types included | Asynchronous | Yes | High | Trained professional(s)/staff |
| 47. Han et al (2008) | Multiple OSG types included | Asynchronous | Not described | Not described | Not described |
| 48. Hargreaves et al (2018) | Bulletin/forum | Asynchronous | Yes | Low | Trained professional(s)/staff |
| 49. Harkin et al (2020) | Multiple OSG types included | Not specified | Not described | Not described | Not described |
| 50. Holdren et al (2023) | Social media/networking website | Asynchronous | Yes – in one or more included interventions or intervention groups | High | Lay peer(s) |
| 51. Holtz et al (2024) | Video chat/video conferencing software | Synchronous | Yes | Low | Trained professional(s)/staff |
| 52. Hong et al (2012) | Multiple OSG types included | Both | Yes – in one or more included interventions or intervention groups | Low | Healthcare professional(s) in one or more included interventions or intervention groups |
| 53. Houlihan and Tariman (2017) | Multiple OSG types included | Not specified | Yes – in one or more included interventions or intervention groups | Low | Not described |
| 54. Hoybye et al (2010) | Multicomponent website | Both | Yes | High | Combination – trained professional(s)/staff and peer facilitator(s) |
| 55. Hoybye et al (2005) | Mailing list | Asynchronous | Not described | Not described | Not described |
| 56. Im (2011) | Multiple OSG types included | Both | Not described | Not described | Not described |
| 57. Im and Chee (2008) | Not specified | Not specified | Not described | Not described | Not described |
| 58. Im et al (2016) | Not specified | Not specified | Not described | Not described | Not described |
| 59. Im et al (2007) | Not specified | Not specified | Not described | Not described | Not described |
| 60. Im et al (2016) | Multiple OSG types included | Asynchronous | Not described | Not described | Not described |
| 61. Im et al (2007) | Not specified | Not specified | Yes – in one or more included interventions or intervention groups | Low | Healthcare professional(s) in one or more included interventions or intervention groups |
| 62. Im et al (2010) | Not specified | Not specified | Not described | Not described | Not described |
| 63. Jo et al (2023) | Bulletin/forum | Asynchronous | Not described | Not described | Not described |
| 64. Kaka et al (2021) | Video chat/video conferencing software | Synchronous | Yes | Low | Healthcare professional(s) in one or more included interventions or intervention groups |
| 65. Kashian and Jacobson (2020) | Social media/networking website | Asynchronous | Not described | Not described | Not described |
| 66. Kim et al (2020) | Multi-component website | Asynchronous | Not described | Not described | Not described |
| 67. Kim et al (2012) | Multi-component website | Asynchronous | Not described | Not described | Not described |
| 68. Kim et al (2017) | Multi-component website | Asynchronous | Not described | Not described | Not described |
| 69. Kim et al (2011) | Multi-component website | Asynchronous | Not described | Not described | Not described |
| 70. Klemm (2012) | Multi-component website | Asynchronous | Yes – in one or more included interventions or intervention groups | High | Healthcare or trained professional(s) |
| 71. Klemm et al (1999) | Not specified | Not specified | Not described | Not described | Not described |
| 72. Klemm et al (2003) | Multiple OSG types included | Asynchronous | Not described | Not described | Not described |
| 73. Lazard et al (2021) | Multiple OSG types included | Both | Not described | Not described | Not described |
| 74. Lazard et al (2021) | Multiple OSG types included | Both | Not described | Not described | Not described |
| 75. Leimeister et al (2008) | Not specified | Not specified | Not described | Not described | Not described |
| 76. Lepore et al (2014) | Multi-component website | Both | Yes | High | Healthcare professional(s) |
| 77. Lepore et al (2019) | Multi-component website | Both | Yes | Low | Healthcare or trained professional(s) |
| 78. Lewallen et al (2014) | Bulletin/forum | Asynchronous | Yes | Low | Not described |
| 79. Lieberman (2004) | Bulletin/forum | Asynchronous | Not described | Not described | Not described |
| 80. Lieberman (2007) | Bulletin/forum | Asynchronous | Yes – in one or more included interventions or intervention groups | Low | Not described |
| 81. Lieberman and Goldstein (2006) | Bulletin/forum | Asynchronous | Not described | Not described | Not described |
| 82. Lieberman et al (2005) | Multiple OSG types included | Both | Yes – in one or more included interventions or intervention groups | Low | Trained professional(s)/staff |
| 83. Lieberman and Russo (2001) | Newsgroup | Not specified | Not described | Not described | Not described |
| 84. Lippka et al (2013) | Bulletin/forum | Asynchronous | Not described | Not described | Not described |
| 85. Lobchuk et al (2015) | Bulletin/forum | Asynchronous | Yes | High | Combination – trained professional(s)/staff and peer facilitator(s) |
| 86. Lovatt et al (2017) | Bulletin/forum | Asynchronous | Yes | High | Trained professional(s)/staff |
| 87. Malloch and Taylor (2019) | Bulletin/forum | Asynchronous | Not described | Not described | Not described |
| 88. Massimi et al (2014) | Multiple OSG types included | Not specified | Yes – in one or more included interventions or intervention groups | Low | Healthcare professional(s) or volunteer(s) |
| 89. McCaughan et al (2017) | Multiple OSG types included | Both | Yes – in one or more included interventions or intervention groups | High | Healthcare or trained professional(s) |
| 90. McLaughlin et al (2012) | Social media/networking website | Asynchronous | Not described | Not described | Not described |
| 91. McLaughlin et al (2016) | Multi-component website | Asynchronous | Not described | Not described | Not described |
| 92. Meier et al (2007) | Mailing list | Asynchronous | Yes | High | Peer facilitators |
| 93. Meng et al (2021) | Multi-component website | Asynchronous | Not described | Not described | Not described |
| 94. Morris et al (2014) | Multiple OSG types included | Asynchronous | Yes – in one or more included interventions or intervention groups | Low | Trained professional(s)/staff |
| 95. Nambisan (2011) | Bulletin/forum | Asynchronous | Not described | Not described | Not described |
| 96. Nambisan (2011) | Bulletin/forum | Asynchronous | Not described | Not described | Not described |
| 97. Namkoong et al (2013) | Multi-component website | Asynchronous | Not described | Not described | Not described |
| 98. Namkoong et al (2017) | Multi-component website | Asynchronous | Not described | Not described | Not described |
| 99. Namkoong et al (2010) | Multi-component website | Asynchronous | Yes | Low | Trained professional(s)/staff |
| 100. Nau (2021) | Video chat/video conferencing | Synchronous | Yes | High | Trained professional(s)/staff |
| 101. Osei et al (2013) | Multi-component website | Asynchronous | Not described | Not described | Not described |
| 102. Owen et al (2004) | Multiple OSG types included | Asynchronous | Not described | Not described | Not described |
| 103. Owen et al (2009) | Multi-component website | Both | Yes – in one or more included interventions or intervention groups | High | Trained professional(s)/staff |
| 104. Owen et al (2015) | Multi-component website | Both | Yes | High | Healthcare or trained professional(s)/staff |
| 105. Owen et al (2005) | Multi-component website | Asynchronous | No | N/A | Not described |
| 106. Painter (2020) | Social media/networking website | Not specified | Not described | Not described | Not described |
| 107. Park et al (2020) | Multi-component website | Asynchronous | Not described | Not described | Not described |
| 108. Preece (2001) | Not specified | Not specified | Not described | Not described | Not described |
| 109. Pyle et al (2021) | Not specified | Not specified | Not described | Not described | Not described |
| 110. Qui et al (2011) | Bulletin/forum | Asynchronous | Not described | Not described | Not described |
| 111. Radin (2006) | Bulletin/forum | Asynchronous | Yes | High | Not described |
| 112. Rains and Meng (2020) | Not specified | Not specified | Not described | Not described | Not described |
| 113. Ray et al (2020) | Pre-recorded videos | Asynchronous | Not described | Not described | Not described |
| 114. Ray et al (2021) | Pre-recorded videos | Asynchronous | Not described | Not described | Not described |
| 115. Rising et al (2017) | Not specified | Not specified | Not described | Not described | Not described |
| 116. Rodgers and Chen (2005) | Bulletin/forum | Asynchronous | Not described | Not described | Not described |
| 117. Rubenstein (2015) | Bulletin/forum | Asynchronous | No | N/A | Not described |
| 118. Salzer et al (2009) | Multi-component website | Not specified | No | N/A | Not described |
| 119. Sandaunet (2008) | Multi-component website | Not specified | Yes | High | Trained professional(s)/staff |
| 120. Sandaunet (2008) | Multi-component website | Not specified | Yes | High | Trained professional(s)/staff |
| 121. Sanders et al (2020) | Bulletin/forum | Asynchronous | Not described | Not described | Not described |
| 122. Sanger et al (2023) | Bulletin/forum | Asynchronous | Yes – in one or more included interventions or intervention groups | Not described | Not described |
| 123. Seale (2006) | Bulletin/forum | Asynchronous | Not described | Not described | Not described |
| 124. Seckin (2009) | Not specified | Not specified | Not described | Not described | Not described |
| 125. Seckin (2013) | Not specified | Not specified | Not described | Not described | Not described |
| 126. Setoyama et al (2011) | Video chat/video conferencing | Asynchronous | Not described | Not described | Not described |
| 127. Seymour-Smith (2013) | Bulletin/forum | Asynchronous | Yes | Low | Not described |
| 128. Sharf (1997) | Mailing list | Asynchronous | No | N/A | Not described |
| 129. Shaw et al (2006) | Multi-component website | Asynchronous | Not described | Not described | Not described |
| 130. Shaw et al (2007) | Multi-component website | Asynchronous | Not described | Not described | Not described |
| 131. Shaw et al (2006) | Multi-component website | Asynchronous | Not described | Not described | Not described |
| 132. Shaw et al (2000) | Multi-component website | Asynchronous | Yes | High | Trained professional(s)/staff |
| 133. Shaw et al (2008) | Multi-component website | Asynchronous | Not described | Not described | Not described |
| 134. Shim et al (2011) | Multi-component website | Asynchronous | Not described | Not described | Not described |
| 135. Sillence and Bussey (2017) | Not specified | Not specified | Yes – in one or more included interventions or intervention groups | Low | Not described |
| 136. Smith et al (2020) | Multi-component website | Not specified | Not described | Not described | Not described |
| 137. Stephen et al (2013) | Chat room/live chat | Synchronous | Yes | High | Healthcare professional(s) |
| 138. Stephen et al (2011) | Not specified | Both | Yes – in one or more included interventions or intervention groups | High | Healthcare professional(s) |
| 139. Street et al (2012) | Not specified | Not specified | Yes | High | Healthcare professional(s) |
| 140. Suarez et al (2024) | Multiple OSG types included | Both | Not described | Not described | Not described |
| 141. Sullivan (2003) | Mailing list | Asynchronous | No | N/A | Not described |
| 142. Turner et al (2001) | Mailing list | Asynchronous | No | N/A | Not described |
| 143. Ure et al (2020) | Video chat/video conferencing | Not specified | Yes – in one or more included interventions or intervention groups | Not described | Not described |
| 144. Valle and Tate (2017) | Social media/networking website | Not specified | Yes | Low | Not described |
| 145. van Uden Kraan et al (2008) | Multi-component website | Asynchronous | Yes – in one or more included interventions or intervention groups | Low | Not described |
| 146. van Uden Kraan et al (2009) | Not specified | Not specified | Not described | Not described | Not described |
| 147. van Uden-Kraan et al (2008) | Not specified | Not specified | Not described | Not described | Not described |
| 148. van Uden-Kraan et al (2011) | Not specified | Not specified | Not described | Not described | Not described |
| 149. Verberne et al (2019) | Bulletin/forum | Asynchronous | Not described | Not described | Not described |
| 150. Vilihauer (2011) | Not specified | Not specified | Yes – in one or more included interventions or intervention groups | Low | Not described |
| 151. Vilihauer (2009) | Mailing list | Asynchronous | Not described | Not described | Not described |
| 152. Vilihauer (2014) | Mailing list | Asynchronous | No | N/A | Not described |
| 153. Wakelin and Street (2015) | Bulletin/forum | Asynchronous | Yes | Low | Trained professional(s)/staff |
| 154. Walsh et al (2024) | Multiple OSG types included | Asynchronous | Not described | Not described | Not described |
| 155. Walther et al (2005) | Multi-component website | Asynchronous | Not described | Not described | Not described |
| 156. Wandrey et al (2016) | Bulletin/forum | Asynchronous | Not described | Not described | Not described |
| 157. Wen et al (2011) | Multi-component website | Asynchronous | Yes | High | Healthcare professional(s) |
| 158. Willard et al (2021) | Video chat/video conferencing | Synchronous | Yes | Low | Health professional(s) |
| 159. Wise et al (2018) | Multi-component website | Asynchronous | Not described | Not described | Not described |
| 160. Wootten et al (2015) | Video chat/video conferencing | Asynchronous | Yes | Low | Not described |
| 161. Xu et al (2014) | Not specified | Not specified | Not described | Not described | Not described |
| 162. Yang et al (2019) | Multi-component website | Not specified | Not described | Not described | Not described |
| 163. Yli-Uotila et al (2013) | Bulletin/forum | Asynchronous | Not described | Not described | Not described |
| 164. Yoo et al (2014) | Multi-component website | Asynchronous | Yes | Low | Trained professional(s)/staff |
| 165. Yoo et al (2013) | Multi-component website | Asynchronous | Not described | Not described | Not described |
| 166. Yoo et al (2014) | Multi-component website | Asynchronous | Yes | Low | Not described |
| 167. Zhou et al (2020) | Chat room/live chat | Synchronous | Not described | Not described | Not described |
| 168. Zhu et al (2021) | Not specified | Not specified | Not described | Not described | Not described |

**^a^Details of moderation description:** Articles were considered low detail if they mentioned the presence of a moderator and/or who moderators were, but no other information. Articles were high detail if they described the role of moderators and how they were involved in the OSG(s).

**References**

1. Abioye O, Kiel L, Kaufman R, Florez N. Cancer health disparities in minority communities: peer support networks can bridge the gap. Cancer Causes Control. 2024 Nov;35(11):1407–11.
2. Adikari A, Silva D, Ranasinghe WKB, Bandaragoda T, Alahakoon O, Persad R, et al. Can online support groups address psychological morbidity of cancer patients? An artificial intelligence based investigation of prostate cancer trajectories. PLOS ONE. 2020 Mar 03;15(3):e0229361. [DOI: 10.1371/journal.pone.0229361]
3. Ahn J, Lee KE. Experiences of peer support activities and the need for a metaverse-based program in young women with breast cancer: A qualitative study. Asia Pac J Oncol Nurs. 2023 Jul;10(7):100253.
4. Algtewi E, Owens J, Baker SR. Online support groups for head and neck cancer and health-related quality of life. Qual Life Res. 2017 Sep 01;26(9):2351–62. [DOI: 10.1007/s11136-017-1575-8]
5. Baker TB, Hawkins R, Pingree S, Roberts LJ, McDowell HE, Shaw BR, et al. Optimizing eHealth breast cancer interventions: which types of eHealth services are effective? Translational Behavioral Medicine. 2011 Mar 1;1(1):134–45. [DOI: 10.1007/s13142-010-0004-0]
6. Batenburg A, Das E. Emotional Approach Coping and the Effects of Online Peer-Led Support Group Participation Among Patients With Breast Cancer: A Longitudinal Study. J Med Internet Res. 2014 Nov 28;16(11):e256. [DOI: 10.2196/jmir.3517]
7. Batenburg A, Das E. Emotional Coping Differences Among Breast Cancer Patients From an Online Support Group: A Cross-Sectional Study. Journal of Medical Internet Research. 2014 Feb 5;16(2):e2831. [DOI: 10.2196/jmir.2831]
8. Baxter J. ‘Keep strong, remember everything you have learnt’: Constructing support and solidarity through online interaction within a UK cancer support group. Discourse & Society. 2018 Jul 1;29(4):363–79. [DOI: 10.1177/09579265187544]
9. Beatty L, Scott K. Examining eHealth use as a coping strategy for cancer-adjustment: An application of the Chronic Illness Model. E-Journal of Applied Psychology. 2013;9(1):9–18. [DOI: 10.7790/ejap.v9i1.349]
10. Bender JL, Katz J, Ferris LE, Jadad AR. What is the role of online support from the perspective of facilitators of face-to-face support groups? A multi-method study of the use of breast cancer online communities. Patient Education and Counseling. 2013 Dec 1;93(3):472–9. [DOI: 10.1016/j.pec.2013.07.009]
11. Broom A. The eMale: Prostate cancer, masculinity and online support as a challenge to medical expertise. Journal of Sociology. 2005 Mar 1;41(1):87–104. [DOI: 10.1177/1440783305050965]
12. Broom A. Virtually He@lthy: The Impact of Internet Use on Disease Experience and the Doctor-Patient Relationship. Qual Health Res. 2005 Mar 1;15(3):325–45. [DOI: 10.1177/1049732304272916]
13. Cabling ML, Turner JW, Hurtado-de-Mendoza A, Zhang Y, Jiang X, Drago F, et al. Sentiment Analysis of an Online Breast Cancer Support Group: Communicating about Tamoxifen. Health Communication. 2018 Sep 2;33(9):1158–65. [DOI: 10.1080/10410236.2017.1339370]
14. Changrani J, Lieberman M, Golant M, Rios P. Online cancer support groups: experiences with underserved immigrant Latinas. Primary Psychiatry. 2008 Oct;15(10):55–62.
15. Chen Z, Koh PW, Ritter PL, Lorig K, Bantum EO, Saria S. Dissecting an Online Intervention for Cancer Survivors: Four Exploratory Analyses of Internet Engagement and Its Effects on Health Status and Health Behaviors. Health Educ Behav. 2015 Feb 1;42(1):32–45. [DOI: 10.1177/1090198114550822]
16. Chiu YC, Hsieh YL. Communication online with fellow cancer patients: Writing to be remembered, gain strength, and find survivors. J Health Psychol. 2013 Dec 1;18(12):1572–81. [DOI: 10.1177/1359105312465915]
17. Crook B, Love B. Examining the Light and Dark of an Online Young Adult Cancer Support Community. Qual Health Res. 2917 May 1;27(6):938–48. [DOI: 10.1177/1049732316672638]
18. Crook B, Glowacki EM, Love B, Jones BL, Macpherson CF, Johnson RH. Hanging by a thread: exploring the features of nonresponse in an online young adult cancer survivorship support community. J Cancer Surviv. 2016 Feb 1;10(1):185–93. [DOI: 10.1007/s11764-015-0465-8]
19. Dickerson SS, Boehmke M, Ogle C, Brown JK. Seeking and managing hope: patients’ experiences using the Internet for cancer care. Oncol Nurs Forum. 2006 Jan 1;33(1):E8-17. [DOI: 10.1188/06.ONF.E8-E17]
20. Dickerson SS, Reinhart A, Boemhke M, Akhu-Zaheya L. Cancer as a Problem to Be Solved: Internet Use and Provider Communication by Men With Cancer. CIN: Computers, Informatics, Nursing. 2011 Jul;29(7):388. [DOI: 10.1097/NCN.0b013e3181f9ddb1]
21. Dolce MC. The Internet as a source of health information: experiences of cancer survivors and caregivers with healthcare providers. Oncol Nurs Forum. 2011 May;38(3):353–9. [DOI: 10.1188/11.ONF.353-359]
22. Donovan EE, LeFebvre L, Tardif S, Brown LE, Love B. Patterns of Social Support Communicated in Response to Expressions of Uncertainty in an Online Community of Young Adults with Cancer. Journal of Applied Communication Research. 2014 Oct 2;42(4):432–55. [DOI: 10.1080/00909882.2014.929725]
23. Donovan E, Martin SR, Seidman LC, Zeltzer LK, Cousineau TM, Payne LA, et al. A Mobile-Based Mindfulness and Social Support Program for Adolescents and Young Adults With Sarcoma: Development and Pilot Testing. JMIR mHealth and uHealth. 2019 Mar 18;7(3):e10921. [DOI: 10.2196/10921]
24. Durant KT, McCray AT, Safran C. Identifying gender-preferred communication styles within online cancer communities: a retrospective, longitudinal analysis. PLoS One. 2012;7(11):e49169. [DOI: 10.1371/journal.pone.0049169]
25. Erfani SS, Blount Y, Abedin B. The influence of health-specific social network site use on the psychological well-being of cancer-affected people. Journal of the American Medical Informatics Association. 2016 May 1;23(3):467–76. [DOI: 10.1093/jamia/ocv170]
26. Erfani SS, Abedin B, Blount Y. The effect of social network site use on the psychological well-being of cancer patients. Journal of the Association for Information Science and Technology. 2017;68(5):1308–22.
27. Eysenbach G. The Impact of the Internet on Cancer Outcomes. CA: A Cancer Journal for Clinicians. 2003;53(6):356–71. [DOI: 10.3322/canjclin.53.6.356]
28. Falisi AL, Wiseman KP, Gaysynsky A, Scheideler JK, Ramin DA, Chou W ying S. Social media for breast cancer survivors: a literature review. J Cancer Surviv. 2017 Dec 1;11(6):808–21. [DOI: 10.1007/s11764-017-0620-5]
29. Fallon EA, Driscoll D, Smith TS, Richardson K, Portier K. Description, characterization, and evaluation of an online social networking community: the American Cancer Society’s Cancer Survivors Network®. J Cancer Surviv. 2018 Oct 1;12(5):691–701. [DOI: 10.1007/s11764-018-0706-8]
30. Feng X, Hu Y, Pfaff H, Liu S, Wang H, Qi Z. The determinants of help-seeking behaviors among cancer patients in online health communities: Evidence from China. Int J Med Inform. 2025 Mar;195:105767.
31. Fogel J, Ribisl KM, Lyons EJ, Morgan PD, Humphreys K. The Underrepresentation of African Americans in Online Cancer Support Groups. Journal of the National Medical Association. 2008 Jun 1;100(6):705–12. [DOI: 10.1016/S0027-9684(15)31346-8]
32. Foster C, Roffe L. An exploration of the internet as a self-management resource. Journal of Research in Nursing. 2009 Jan 1;14(1):13–24. [DOI: 10.1177/1744987108099237]
33. Frost J, Vermeulen IE, Beekers N. Anonymity Versus Privacy: Selective Information Sharing in Online Cancer Communities. J Med Internet Res. 2014 May 14;16(5):e126. [DOI: 10.2196/jmir.2684]
34. Garg R, Rebić N, De Vera MA. Information Needs About Cancer Treatment, Fertility, and Pregnancy: Qualitative Descriptive Study of Reddit Threads. JMIR Cancer. 2020 Dec 2;6(2):e17771. [DOI: 10.2196/17771]
35. Gill PS, Whisnant B. A qualitative assessment of an online support community for ovarian cancer patients. PROM. 2012 Oct 4;3:51–8. [DOI: 10.2147/PROM.S36034]
36. Ginossar T. Online participation: a content analysis of differences in utilization of two online cancer communities by men and women, patients and family members. Health Commun. 2008;23(1):1–12. [DOI: 10.1080/10410230701697100]
37. Gits AA, Ritter PL, Plant K, Lorig K. Coding and evaluating facilitator posts for an online cancer survivor workshop. J Psychosoc Oncol. 2013;31(2):219–34. [DOI: 10.1080/07347332.2012.761321]
38. Gooden RJ, Winefield HR. Breast and Prostate Cancer Online Discussion Boards: A Thematic Analysis of Gender Differences and Similarities. J Health Psychol. 2007 Jan 1;12(1):103–14. [DOI: 10.1177/1359105307071744]
39. Gorlick A, Bantum EO, Owen JE. Internet-based interventions for cancer-related distress: exploring the experiences of those whose needs are not met. Psychooncology. 2014 Apr;23(4):452–8. [DOI: 10.1002/pon.3443]
40. Griffiths KM, Calear AL, Banfield M. Systematic Review on Internet Support Groups (ISGs) and Depression (1): Do ISGs Reduce Depressive Symptoms? J Med Internet Res. 2009 Sep 30;11(3):e40. [DOI: 10.2196/jmir.1270]
41. Griffiths C, Panteli N, Brunton D, Marder B, Williamson H. Designing and evaluating the acceptability of Realshare: An online support community for teenagers and young adults with cancer. J Health Psychol. 2015 Dec 1;20(12):1589–601. [DOI: 10.1177/1359105313519154]
42. Gupta T, Schapira L. Online Communities as Sources of Peer Support for People Living With Cancer: A Commentary. JOP. 2018 Dec;14(12):725–30. [DOI: 10.1200/JOP.18.0026]
43. Han JY, Kim JH, Yoon HJ, Shim M, McTavish FM, Gustafson DH. Social and Psychological Determinants of Levels of Engagement with an Online Breast Cancer Support Group: Posters, Lurkers, and Non-Users. J Health Commun. 2012;17(3):356–71. [DOI: 10.1080/10810730.2011.585696]
44. Han JY, Hou J, Kim E, Gustafson DH. Lurking as an active participation process: a longitudinal investigation of engagement with an online cancer support group. Health Commun. 2014;29(9):911–23. [DOI: 10.1080/10410236.2013.816911]
45. Han JY, Kim E, Lee YI, Shah DV, Gustafson DH. A Longitudinal Investigation of Empathic Exchanges in Online Cancer Support Groups: Message Reception and Expression Effects on Patients’ Psychosocial Health Outcomes. J Health Commun. 2019;24(6):615–23. [DOI: 10.1080/10810730.2019.1644401]
46. Han JY, Shah DV, Kim E, Namkoong K, Lee SY, Moon TJ, et al. Empathic Exchanges in Online Cancer Support Groups: Distinguishing Message Expression and Reception Effects. Health Commun. 2011 Mar;26(2):185–97. [DOI: 10.1080/10410236.2010.544283]
47. Han JY, Shaw BR, Hawkins RP, Pingree S, McTavish F, Gustafson DH. Expressing Positive Emotions within Online Support Groups by Women with Breast Cancer. J Health Psychol. 2008 Nov;13(8):1002–7. [DOI: 10.1177/1359105308097963]
48. Hargreaves S, Bath PA, Duffin S, Ellis J. Sharing and Empathy in Digital Spaces: Qualitative Study of Online Health Forums for Breast Cancer and Motor Neuron Disease (Amyotrophic Lateral Sclerosis). Journal of Medical Internet Research. 2018 Jun 14;20(6):e9709. [DOI: 10.2196/jmir.9709]
49. Harkin LJ, Beaver K, Dey P, Choong KA. Secret groups and open forums: Defining online support communities from the perspective of people affected by cancer. DIGITAL HEALTH. 2020 Jan 1;6:2055207619898993. [DOI:10.1177/2055207619898993]
50. Holdren J, Surkan K, Downing A. Perspectives of People With Cancer or Hereditary Cancer Risk on the Use and Value of Online Peer Support. J Patient Cent Res Rev. 2023;10(2):58–67.
51. Holtz BE, Mitchell KM, Strand D, Hirko K. Perceptions of Telehealth-Based Cancer Support Groups at a Rural Community Oncology Program. J Cancer Educ. 2024 Aug;39(4):418–25.
52. Hong Y, Peña-Purcell NC, Ory MG. Outcomes of online support and resources for cancer survivors: A systematic literature review. Patient Education and Counseling. 2012 Mar 1;86(3):288–96. [DOI: 10.1016/j.pec.2011.06.014]
53. Houlihan MC, Tariman JD. Comparison of Outcome Measures for Traditional and Online Support Groups for Breast Cancer Patients: An Integrative Literature Review. J Adv Pract Oncol. 2017;8(4):348–59. [DOI: 10.6004/jadpro.2017.8.4.4]
54. Høybye MT, Dalton SO, Christensen J, Ross L, Kuhn KG, Johansen C. Social and psychological determinants of participation in internet-based cancer support groups. Support Care Cancer. 2010 May 1;18(5):553–60. [DOI: 10.1007/s00520-009-0683-6]
55. Høybye MT, Johansen C, Tjørnhøj-Thomsen T. Online interaction. Effects of storytelling in an internet breast cancer support group. Psycho-Oncology. 2005;14(3):211–20. [DOI: 10.1002/pon.837]
56. Im EO. Online Support of Patients and Survivors of Cancer. Seminars in Oncology Nursing. 2011 Aug 1;27(3):229–36. [DOI: 10.1016/j.soncn.2011.04.008]
57. Im EO, Chee W. The Use of Internet Cancer Support Groups by Ethnic Minorities. J Transcult Nurs. 2008 Jan 1;19(1):74–82. [DOI: 10.1177/1043659607309140]
58. Im EO, Lee B, Chee W. The Use of Internet Cancer Support Groups by Asian Americans and White Americans Living With Cancer. J Transcult Nurs. 2011 Oct 1;22(4):386–96.
59. Im EO, Chee W, Liu Y, Lim HJ, Guevara E, Tsai HM, et al. Characteristics of Cancer Patients in Internet Cancer Support Groups. Comput Inform Nurs. 2007;25(6):334–43.
60. Im EO, Ji X, Zhang J, Kim S, Lee Y, Chee E, et al. Issues in Developing and Evaluating a Culturally Tailored Internet Cancer Support Group. CIN: Computers, Informatics, Nursing. 2016 Oct;34(10):462. [DOI: 10.1097/CIN.0000000000000261]
61. Im EO, Chee W, Lim HJ, Liu Y, Guevara E, Kim KS. Patients’ attitudes toward internet cancer support groups. Oncol Nurs Forum. 2007 May;34(3):705–12. [DOI: 10.1188/07.ONF.705-712] [DOI: 10.1097/NCC.0b013e3181c8e5d5]
62. Im EO, Lee B, Chee W. Shielded From the Real World: Perspectives on Internet Cancer Support Groups by Asian Americans. Cancer Nursing. 2010 Jun;33(3):E10. [DOI: 10.1097/NCC.0b013e3181c8e5d5]
63. Jo W, Jang SH, Shin EK. Stage distinctive communication networks of the online breast cancer community. Sci Rep. 2023 Jan 31;13(1):1726.
64. Kaka A, Shama W, Lucchetta S, Nishimura C, Cottingham D, Positano K, et al. Virtual support groups in pediatric hematology/oncology during COVID-19: Lessons learned from the SickKids experience. Pediatric Blood & Cancer. 2021;68(12):e29378. [DOI: 10.1002/pbc.29378]
65. Kashian N, Jacobson S. Factors of Engagement and Patient-Reported Outcomes in a Stage IV Breast Cancer Facebook Group. Health Commun. 2020 Jan;35(1):75–82. [DOI: 10.1080/10410236.2018.1536962]
66. Kim SC, Hawkins RP, Shah DV, Gustafson DH, Baker TB. Understanding how e-health interventions meet psychosocial needs of breast cancer patients: The pathways of influence on quality of life and cancer concerns. Psycho-Oncology. 2020;29(10):1704–12. [DOI: 10.1002/pon.5512]
67. Kim E, Han JY, Moon TJ, Shaw B, Shah DV, McTavish FM, et al. The Process and Effect of Supportive Message Expression and Reception in Online Breast Cancer Support Groups. Psychooncology. 2012 May;21(5):531–40. [DOI: 10.1002/pon.1942]
68. Kim E, Scheufele DA, Han JY, Shah D. Opinion Leaders in Online Cancer Support Groups: An Investigation of Their Antecedents and Consequences. Health Commun. 2017 Feb;32(2):142–51. [DOI: 10.1080/10410236.2015.1110005]
69. Kim E, Han JY, Shah D, Shaw B, McTavish F, Gustafson DH, et al. Predictors of Supportive Message Expression and Reception in an Interactive Cancer Communication System. Journal of Health Communication. 2011 Nov 1;16(10):1106–21. [DOI: 10.1080/10810730.2011.571337]
70. Klemm P. Effects of Online Support Group Format (Moderated vs Peer-Led) on Depressive Symptoms and Extent of Participation in Women With Breast Cancer. CIN: Computers, Informatics, Nursing. 2012 Jan;30(1):9. [DOI: 10.1097/NCN.0b013e3182343efa]
71. Klemm P, Hurst M, Dearholt SL, Trone SR. Gender differences on Internet cancer support groups. Comput Nurs. 1999;17(2):65–72. [PMID: 10194883]
72. Klemm P, Bunnell D, Cullen M, Soneji R, Gibbons P, Holecek A. Online Cancer Support Groups: A Review of the Research Literature. CIN: Computers, Informatics, Nursing. 2003 Jun;21(3):136. [DOI: 10.1097/00024665-200305000-00010
73. Lazard AJ, Collins MKR, Hedrick A, Varma T, Love B, Valle CG, et al. Using Social Media for Peer-to-Peer Cancer Support: Interviews With Young Adults With Cancer. JMIR Cancer. 2021 Sep 2;7(3):e28234. [DOI: 10.2196/28234]
74. Lazard AJ, Collins MKR, Hedrick A, Horrell LN, Varma T, Love B, et al. Initiation and changes in use of social media for peer support among young adult cancer patients and survivors. Psycho-Oncology. 2021;30(11):1859–65. [DOI: 10.1002/pon.5758]
75. Leimeister JM, Schweizer K, Leimeister S, Krcmar H. Do virtual communities matter for the social support of patients? Antecedents and effects of virtual relationships in online communities. Information Technology & People. 2008;21(4):350–74. [DOI: 10.1108/09593840810919671]
76. Lepore SJ, Buzaglo JS, Lieberman MA, Golant M, Greener JR, Davey A. Comparing Standard Versus Prosocial Internet Support Groups for Patients With Breast Cancer: A Randomized Controlled Trial of the Helper Therapy Principle. J Clin Oncol. 2014 Dec 20;32(36):4081–6. [DOI:  10.1200/JCO.2014.57.0093]
77. Lepore SJ, Rincon MA, Buzaglo JS, Golant M, Lieberman MA, Bauerle Bass S, et al. Digital literacy linked to engagement and psychological benefits among breast cancer survivors in Internet-based peer support groups. European Journal of Cancer Care. 2019;28(4):e13134. [DOI: 10.1111/ecc.13134]
78. Lewallen AC, Owen JE, Bantum EO, Stanton AL. How language affects peer responsiveness in an online cancer support group: implications for treatment design and facilitation. Psycho-Oncology. 2014;23(7):766–72. [DOI: 10.1002/pon.3477]
79. Lieberman M. Self-management in online self-help groups for breast cancer patients: Finding the right group, a speculative hypothesis. International Journal of Self Help and Self Care. 2004 Aug 6;2(4):313–28. [DOI: 10.2190/0009-9e12-ht6d-3j13]
80. Lieberman M. The role of insightful disclosure in outcomes for women in peer-directed breast cancer groups: a replication study. Psycho-Oncology. 2007;16(10):961–4. [DOI: 10.1002/pon.1181]
81. Lieberman MA, Goldstein BA. Not all negative emotions are equal: the role of emotional expression in online support groups for women with breast cancer. Psycho-Oncology. 2006;15(2):160–8. [DOI: 10.1002/pon.932]
82. Lieberman M, Golant M, Winzelberg A, Mctavish F. Comparisons: Professionally-directed and self-directed internet groups for women with breast cancer. International Journal for Self Help and Self Care. 2005 Mar;2(3):219–35. [DOI: 10.2190/ge85-j31w-xjv7-lb9l]
83. Lieberman M, Russo S. Self help groups and the internet: Breast cancer newsgroups. International Journal of Self Help and Self Care. 2001 Jan 1;1(4):323–44. [DOI: 10.2190/egwj-gbqd-kpda-7uud]
84. Lippka Y, Patschan O, Todenhöfer T, Schwentner C, Gutzeit A, Merseburger AS, et al. Bladder cancer discussed on the internet: a systematic analysis of gender differences of initial posters on an online discussion board. SpringerPlus. 2013 Sep 8;2(1):445. [DOI: 10.1186/2193-1801-2-445]
85. Lobchuk M, McClement S, Rigney M, Copeland A, Bayrampour H. A Qualitative Analysis of “Naturalistic” Conversations in a Peer-Led Online Support Community for Lung Cancer. Cancer Nursing. 2015 Oct;38(5):E21. [DOI: 10.1097/NCC.0000000000000207]
86. Lovatt M, Bath PA, Ellis J. Development of Trust in an Online Breast Cancer Forum: A Qualitative Study. Journal of Medical Internet Research. 2017 May 23;19(5):e7471. [DOI: 10.2196/jmir.7471]
87. Malloch YZ, Taylor LD. Emotional Self-Disclosure in Online Breast Cancer Support Groups: Examining Theme, Reciprocity, and Linguistic Style Matching. Health Commun. 2019 Jun;34(7):764–73. [DOI: 10.1080/10410236.2018.1434737]
88. Massimi M, Bender JL, Witteman HO, Ahmed OH. Life transitions and online health communities: reflecting on adoption, use, and disengagement. In: Proceedings of the 17th ACM conference on Computer supported cooperative work & social computing [Internet]. New York, NY, USA: Association for Computing Machinery; 2014. p. 1491–501. (CSCW ’14). [DOI: 10.1145/2531602.2531622]
89. McCaughan E, Parahoo K, Hueter I, Northouse L, Bradbury I. Online support groups for women with breast cancer. Cochrane Database Syst Rev. 2017 Mar 10 3(3): CD011652. [DOI: 10.1002/14651858.CD011652.pub2]
90. McLaughlin M, Nam Y, Gould J, Pade C, Meeske KA, Ruccione KS, et al. A videosharing social networking intervention for young adult cancer survivors. Computers in Human Behavior. 2012 Mar 1;28(2):631–41. [DOI: 10.1016/j.chb.2011.11.009]
91. Mclaughlin B, Yang J, Yoo W, Shaw B, Kim SY, Shah D, et al. The Effects of Expressing Religious Support Online for Breast Cancer Patients. Health Commun. 2016;31(6):762–71. [10.1080/10410236.2015.1007550]
92. Meier A, Lyons EJ, Frydman G, Forlenza M, Rimer BK. How Cancer Survivors Provide Support on Cancer-Related Internet Mailing Lists. J Med Internet Res. 2007 May 14;9(2):e12. [DOI: 10.2196/jmir.9.2.e12]
93. Meng J, Rains SA, An Z. How Cancer Patients Benefit from Support Networks Offline and Online: Extending the Model of Structural-to-Functional Support. Health Commun. 2021 Feb;36(2):198–206. [DOI: 10.1080/10410236.2019.1673947]
94. Morris BA, Lepore SJ, Wilson B, Lieberman MA, Dunn J, Chambers SK. Adopting a survivor identity after cancer in a peer support context. J Cancer Surviv. 2014 Sep 1;8(3):427–36. [DOI: 10.1007/s11764-014-0355-5]
95. Nambisan P. Evaluating patient experience in online health communities: Implications for health care organizations. Health Care Management Review. 2011 Jun;36(2):124. [DOI: 10.1097/HMR.0b013e3182099f82]
96. Nambisan P. Information seeking and social support in online health communities: impact on patients’ perceived empathy. Journal of the American Medical Informatics Association. 2011 May 1;18(3):298–304. [DOI: 10.1136/amiajnl-2010-000058]
97. Namkoong K, McLaughlin B, Yoo W, Hull SJ, Shah DV, Kim SC, et al. The Effects of Expression: How Providing Emotional Support Online Improves Cancer Patients’ Coping Strategies. JNCI Monographs. 2013 Dec 1;2013(47):169–74. [DOI: 10.1093/jncimonographs/lgt033]
98. Namkoong K, Shah DV, Gustafson DH. Offline Social Relationships and Online Cancer Communication: Effects of Social and Family Support on Online Social Network Building. Health Communication. 2017 Nov 2;32(11):1422–9. [DOI: 10.1080/10410236.2016.1230808]
99. Namkoong K, Shah DV, Han JY, Kim SC, Yoo W, Fan D, et al. Expression and reception of treatment information in breast cancer support groups: how health self-efficacy moderates effects on emotional well-being. Patient Educ Couns. 2010 Dec;81 Suppl:S41-47. [DOI: 10.1016/j.pec.2010.09.009]
100. Nau E. An online group for young women with breast cancer: sparking online engagement. Social Work With Groups. 2022 Oct 2;45(3–4):257–67. [DOI: 10.1080/01609513.2021.1907996]
101. Osei DK, Lee JW, Modest NN, Pothier PKT. Effects of an online support group for prostate cancer survivors: a randomized trial. Urol Nurs. 2013;33(3):123–33. [DOI: 10.7257/1053-816X.2013.33.3.123]
102. Owen JE, Klapow JC, Roth DL, Tucker DC. Use of the Internet for Information and Support: Disclosure Among Persons with Breast and Prostate Cancer. J Behav Med. 2004 Oct 1;27(5):491–505. [DOI: 10.1023/B:JOBM.0000047612.81370.f7]
103. Owen JE, Bantum EO, Golant M. Benefits and challenges experienced by professional facilitators of online support groups for cancer survivors. Psycho-Oncology. 2009;18(2):144–55. [DOI: 10.1002/pon.1374]
104. Owen JE, Bantum EO, Gorlick A, Stanton AL. Engagement with a Social Networking Intervention for Cancer-Related Distress. Annals of Behavioral Medicine. 2015 Apr 1;49(2):154–64. [DOI: 10.1007/s12160-014-9643-6]
105. Owen JE, Klapow JC, Roth DL, Shuster JL Jr, Bellis J, Meredith R, et al. Randomized pilot of a self-guided internet coping group for women with early-stage breast cancer. Annals of Behavioral Medicine. 2005 Aug 1;30(1):54–64. [DOI: 10.1207/s15324796abm3001_7]
106. Painter C. Reaching for the Brass Ring. Cell. 2020 Apr 2;181(1):8–14. [DOI: 10.1016/j.cell.2020.02.030]
107. Park KA, Eum SY, Oh H, Cho MH, Chang HS, Lee YS, et al. Factors affecting online health community participation behavior in patients with thyroid cancer. PLoS One. 2020 Jun 24;15(6):e0235056. [DOI: 10.1371/journal.pone.0235056]
108. Preece J. Sociability and usability in online communities: Determining and measuring success. Behaviour & Information Technology. 2001 Jan 1;20(5):347–56. [DOI: 10.1080/01449290110084683]
109. Pyle D, Tehan G, Lamont-Mills A, Chambers SK. Exploring predictors of and barriers to online prostate cancer community use: A cross-sectional survey of users and non-users. Psycho-Oncology. 2022;31(5):824–31. [DOI: 10.1002/pon.5867]
110. Qiu B, Zhao K, Mitra P, Wu D, Caragea C, Yen J, et al. Get Online Support, Feel Better -- Sentiment Analysis and Dynamics in an Online Cancer Survivor Community. 2011 IEEE Third Int’l Conference on Privacy, Security, Risk and Trust and 2011 IEEE Third Int’l Conference on Social Computing. 2011 Oct;274–81. [DOI: 10.1109/PASSAT/SocialCom.2011.127]
111. Radin P. “To me, it’s my life”: Medical communication, trust, and activism in cyberspace. Social Science & Medicine. 2006 Feb 1;62(3):591–601. [DOI: 10.1016/j.socscimed.2005.06.022]
112. Rains SA, Meng J. Social Enhancement and Compensation in Online Social Support among Cancer Patients: The Role of Social Network Properties. Health Communication. 2022 Mar 21;37(4):490–7. [DOI: 10.1080/10410236.2020.1853327]
113. Ray CD, Floyd K, Tietsort CJ, Veluscek AM, Otmar CD, Hashi EC, et al. Mixed Messages: I. The Consequences of Communicating Negative Statements Within Emotional Support Messages to Cancer Patients. J Patient Exp. 2020 Aug;7(4):593–9. [DOI: 10.1177/2374373519873781]
114. Ray CD, Harvey J, Floyd K, Bonito JA, Reblin M. Mixed Messages: II. Outcomes Associated with the Proportion and Placement of Negative Statements in Support Messages. Health Communication. 2021 Jun 7;36(7):856–65. [DOI: 10.1080/10410236.2020.1719322]
115. Rising CJ, Bol N, Burke-Garcia A, Rains S, Wright KB. Perceived Stress in Online Prostate Cancer Community Participants: Examining Relationships with Stigmatization, Social Support Network Preference, and Social Support Seeking. Journal of Health Communication. 2017 Jun 3;22(6):469–76. [DOI: 10.1080/10810730.2017.1304471]
116. Rodgers S, Chen Q. Internet Community Group Participation: Psychosocial Benefits for Women with Breast Cancer. Journal of Computer-Mediated Communication. 2005 Jul 1;10(4):JCMC1047. [DOI: 10.1111/j.1083-6101.2005.tb00268.x]
117. Rubenstein EL. “They are always there for me”: The convergence of social support and information in an online breast cancer community. Journal of the Association for Information Science and Technology. 2015;66(7):1418–30. [DOI: 10.1002/asi.23263]
118. Salzer MS, Palmer SC, Kaplan K, Brusilovskiy E, Ten Have T, Hampshire M, et al. A randomized, controlled study of Internet peer-to-peer interactions among women newly diagnosed with breast cancer. Psycho-Oncology. 2010;19(4):441–6. [DOI: 10.1002/pon.1586]
119. Sandaunet AG. A Space for Suffering? Communicating Breast Cancer in an Online Self-Help Context. Qual Health Res. 2008 Dec 1;18(12):1631–41. [DOI: 10.1177/1049732308327076]
120. Sandaunet AG. The challenge of fitting in: non-participation and withdrawal from an online self-help group for breast cancer patients. Sociology of Health & Illness. 2008;30(1):131–44. [DOI: 10.1111/j.1467-9566.2007.01041.x]
121. Sanders R, Araujo TB, Vliegenthart R, Eenbergen MC van, Weert JC van, Linn AJ. Patients’ Convergence of Mass and Interpersonal Communication on an Online Forum: Hybrid Methods Analysis. Journal of Medical Internet Research. 2020 Oct 19;22(10):e18303. [DOI: 10.2196/18303]
122. Sanger S, Duffin S, Gough RE, Bath PA. Use of Online Health Forums by People Living With Breast Cancer During the COVID-19 Pandemic: Thematic Analysis. JMIR Cancer. 2023 Feb 7;9:e42783.
123. Seale C. Gender accommodation in online cancer support groups. Health. 2006;10(3):345–60. [DOI: 10.1177/1363459306064495]
124. Seçkin G. Internet Technology in Service of Personal Health Care Management: Patient Perspective. Journal of Technology in Human Services. 2009 Apr 23;27(2):79–92. [DOI: 10.1080/15228830902749179]
125. Seçkin G. Satisfaction with health status among cyber patients: testing a mediation model of electronic coping support. Behaviour & Information Technology. 2013 Jan 1;32(1):91–101. [DOI: 10.1080/0144929X.2011.603359]
126. Setoyama Y, Yamazaki Y, Namayama K. Benefits of Peer Support in Online Japanese Breast Cancer Communities: Differences Between Lurkers and Posters. J Med Internet Res. 2011 Dec 29;13(4):e122. [DOI: 10.2196/jmir.1696]
127. Seymour-Smith S. A reconsideration of the gendered mechanisms of support in online interactions about testicular implants: A discursive approach. Health Psychology. 2013;32(1):91–9. [DOI: 10.1037/a0029507]
128. Sharf BF. Communicating Breast Cancer On-Line: Support and Empowerment on the Internet. Women & Health. 1997 Sep 1;26(1):65–84. [DOI: 10.1300/J013v26n01_05]
129. Shaw BR, Hawkins R, McTavish F, Pingree S, Gustafson DH. Effects of Insightful Disclosure Within Computer Mediated Support Groups on Women With Breast Cancer. Health Communication. 2006 Mar 1;19(2):133–42. [10.1207/s15327027hc1902_5]
130. Shaw B, Han JY, Kim E, Gustafson D, Hawkins R, Cleary J, et al. Effects of prayer and religious expression within computer support groups on women with breast cancer. Psycho-Oncology. 2007;16(7):676–87. [DOI: 10.1002/pon.1129]
131. Shaw BR, Hawkins R, Arora N, McTavish F, Pingree S, Gustafson DH. An Exploratory Study of Predictors of Participation in a Computer Support Group for Women With Breast Cancer. CIN: Computers, Informatics, Nursing. 2006 Feb;24(1):18. [DOI: 10.1097/00024665-200601000-00007]
132. Shaw BR, McTavish F, Hawkins R, Gustafson DH, Pingree S. Experiences of Women with Breast Cancer: Exchanging Social Support over the CHESS Computer Network. Journal of Health Communication. 2000;5(2):135-159. [DOI: 10.1080/108107300406866]
133. Shaw BR, Han JY, Hawkins RP, McTavish FM, Gustafson DH. Communicating about Self and Others within an Online Support Group for Women with Breast Cancer and Subsequent Outcomes. J Health Psychol. 2008 Oct 1;13(7):930–9. [DOI: 10.1177/1359105308095067]
134. Shim M, Cappella JN, Han JY. How Does Insightful and Emotional Disclosure Bring Potential Health Benefits?: Study Based on Online Support Groups for Women with Breast Cancer. J Commun. 2011 Jun;61(3):432–64. [DOI: 10.1111/j.1460-2466.2011.01555.x]
135. Sillence E, Bussey L. Changing hospitals, choosing chemotherapy and deciding you’ve made the right choice: Understanding the role of online support groups in different health decision-making activities. Patient Education and Counseling. 2017 May 1;100(5):994–9. [DOI: 10.1016/j.pec.2016.12.004]
136. Smith CE, Levonian Z, Ma H, Giaquinto R, Lein-Mcdonough G, Li Z, et al. “I Cannot Do All of This Alone”: Exploring Instrumental and Prayer Support in Online Health Communities. ACM Trans Comput-Hum Interact. 2020 Aug 17;27(5):38:1-38:41. [DOI: 10.1145/3402855]
137. Stephen J, Collie K, McLeod D, Rojubally A, Fergus K, Speca M, et al. Talking with text: Communication in therapist-led, live chat cancer support groups. Social Science & Medicine. 2014 Mar 1;104:178–86. [DOI: 10.1016/j.socscimed.2013.12.001]
138. Stephen JE, Christie G, Flood K, Golant M, Rahn M, Rennie H, et al. Facilitating online support groups for cancer patients: the learning experience of psycho-oncology clinicians. Psycho-Oncology. 2011;20(8):832–40. [DOI: 10.1002/pon.1791]
139. Street AF, Wakelin K, Hordern A, Bruce N, Horey D. Dignity and Deferral Narratives as Strategies in Facilitated Technology-Based Support Groups for People with Advanced Cancer. Nursing Research and Practice. 2012;2012(1):647836. [DOI: 10.1155/2012/647836]
140. Suarez NRE, Morrow AS, LaVecchia CM, Dugas M, Carnovale V, Maraboto A, et al. Connected and supported: a scoping review of how online communities provide social support for breast cancer survivors. J Cancer Surviv. 2024 Aug 28;
141. Sullivan CF. Gendered Cybersupport: A Thematic Analysis of Two Online Cancer Support Groups. J Health Psychol. 2003 Jan 1;8(1):83–104. [10.1177/1359105303008001446]
142. Turner JW, Grube JA, Meyers J. Developing an optimal match within online communities: an exploration of CMC support communities and traditional support. Journal of Communication. 2001;51(2):231–51. [DOI: 10.1111/j.1460-2466.2001.tb02879.x]
143. Ure C, Cooper-Ryan AM, Condie J, Galpin A. Exploring Strategies for Using Social Media to Self-Manage Health Care When Living With and Beyond Breast Cancer: In-Depth Qualitative Study. J Med Internet Res. 2020 May 25;22(5):e16902. [DOI: 10.2196/16902]
144. Valle CG, Tate DF. Engagement of young adult cancer survivors within a Facebook-based physical activity intervention. Translational Behavioral Medicine. 2017 Dec 1;7(4):667–79. [DOI: 10.1007/s13142-017-0483-3]
145. van Uden-Kraan CF, Drossaert CHC, Taal E, Lebrun CEI, Drossaers-Bakker KW, Smit WM, et al. Coping with somatic illnesses in online support groups: Do the feared disadvantages actually occur? Computers in Human Behavior. 2008 Mar 1;24(2):309–24. [DOI: 10.1016/j.chb.2007.01.014]
146. van Uden-Kraan CF, Drossaert CHC, Taal E, Seydel ER, van de Laar MAFJ. Participation in online patient support groups endorses patients’ empowerment. Patient Education and Counseling. 2009 Jan 1;74(1):61–9. [DOI: 10.1016/j.pec.2008.07.044]
147. van Uden-Kraan CF, Drossaert CHC, Taal E, Shaw BR, Seydel ER, van de Laar MAFJ. Empowering Processes and Outcomes of Participation in Online Support Groups for Patients With Breast Cancer, Arthritis, or Fibromyalgia. Qual Health Res. 2008 Mar 1;18(3):405–17. [DOI: 10.1177/1049732307313429]
148. van Uden-Kraan CF, Drossaert CH, Taal E, Smit WM, Moens HJB, Laar MAV de. Determinants of Engagement in Face-to-Face and Online Patient Support Groups. Journal of Medical Internet Research. 2011 Dec 7;13(4):e1718. [DOI: 10.2196/jmir.1718]
149. Verberne S, Batenburg A, Sanders R, Eenbergen M van, Das E, Lambooij MS. Analyzing Empowerment Processes Among Cancer Patients in an Online Community: A Text Mining Approach. JMIR Cancer. 2019 Apr 17;5(1):e9887. [DOI: 10.2196/cancer.9887]
150. Vilhauer RP. ‘Them’ and ‘us’: The experiences of women with metastatic disease in mixed-stage versus stage-specific breast cancer support groups. Psychology & Health. 2011 Jun 1;26(6):781–97. [DOI: 10.1080/08870446.2010.496853]
151. Vilhauer RP. Perceived Benefits of Online Support Groups for Women with Metastatic Breast Cancer. Women & Health. 2009 Oct 19;49(5):381–404. [DOI: 10.1080/03630240903238719]
152. Vilhauer RP. Computer-mediated and face-to-face communication in metastatic cancer support groups. Palliative & Supportive Care. 2014 Aug;12(4):287–97. [DOI: 10.1017/S1478951513000126]
153. Wakelin K, Street AF. An Online Expressive Writing Group for People Affected by Cancer: A Virtual Third Place. Australian Social Work. 2015 Apr 3;68(2):198–211. [DOI: 10.1080/0312407X.2014.991336]
154. Walsh CA, Currin-McCulloch J, Faris NR, Nguyen TST, Al Achkar M. “Living with Loss”: A qualitative exploration of existential fears among people with advanced lung cancer in online lung cancer support groups. Palliat Support Care. 2024 Feb 29;1–6.
155. Walther JB, Pingree S, Hawkins RP, Buller DB. Attributes of interactive online health information systems. J Med Internet Res. 2005 Jul 1;7(3):e33. [DOI: 10.2196/jmir.7.3.e33]
156. Wandrey RL, Qualls WD, Mosack KE. Are mainstream support services meeting the needs of sexual minority women with breast cancer? An exploration of the perspectives and experiences of users of an online support forum. Journal of Gay & Lesbian Social Services. 2016 Oct 1;28(4):336–48. [DOI: 10.1080/10538720.2016.1221783]
157. Wen KY, McTavish F, Kreps G, Wise M, Gustafson D. From Diagnosis to Death: A Case Study of Coping With Breast Cancer as Seen Through Online Discussion Group Messages. J Comput Mediat Commun. 2011 Jan;16(2):331–61. [DOI: 10.1111/j.1083-6101.2011.01542.x]
158. Willard VW, Brasher S, Harman JL, Jurbergs N. Virtual Group Activities as a New Platform for Socialization in Children With Pediatric Cancer: A Case Series From the COVID-19 Pandemic. Journal of Pediatric Hematology/Oncology. 2022 Nov;44(8):462. [DOI: 10.1097/MPH.0000000000002394]
159. Wise M, Marchand LR, Roberts LJ, Chih MY. Suffering in Advanced Cancer: A Randomized Control Trial of a Narrative Intervention. J Palliat Med. 2018 Feb 1;21(2):200–7. [DOI: 10.1089/jpm.2017.0007]
160. Wootten AC, Abbott JAM, Meyer D, Chisholm K, Austin DW, Klein B, et al. Preliminary Results of a Randomised Controlled Trial of an Online Psychological Intervention to Reduce Distress in Men Treated for Localised Prostate Cancer. European Urology. 2015 Sep 1;68(3):471–9. [DOI: 10.1016/j.eururo.2014.10.024]
161. Xu Y, Testerman LS, Owen JE, Bantum EO, Thornton AA, Stanton AL. Modeling intention to participate in face-to-face and online lung cancer support groups. Psycho-Oncology. 2014;23(5):555–61. [DOI: 10.1002/pon.3449]
162. Yang D, Yao Z, Seering J, Kraut R. The Channel Matters: Self-disclosure, Reciprocity and Social Support in Online Cancer Support Groups. In: Proceedings of the 2019 CHI Conference on Human Factors in Computing Systems [Internet]. New York, NY, USA: Association for Computing Machinery; 2019. p. 1–15. (CHI ’19). [DOI: 10.1145/3290605.3300261]
163. Yli-Uotila T, Rantanen A, Suominen T. Motives of cancer patients for using the internet to seek social support. European Journal of Cancer Care. 2013;22(2):261–71. [DOI: 10.1111/ecc.12025]
164. Yoo W, Namkoong K, Choi M, Shah DV, Tsang S, Hong Y, et al. Giving and receiving emotional support online: Communication competence as a moderator of psychosocial benefits for women with breast cancer. Computers in Human Behavior. 2014 Jan 1;30:13–22. [DOI: 10.1016/j.chb.2013.07.024]
165. Yoo W, Chih MY, Kwon MW, Yang J, Cho E, McLaughlin B, et al. Predictors of the change in the expression of emotional support within an online breast cancer support group: A longitudinal study. Patient Education and Counseling. 2013 Jan 1;90(1):88–95. [DOI: 10.1016/j.pec.2012.10.001]
166. Yoo W, Shah DV, Shaw BR, Kim E, Smaglik P, Roberts LJ, et al. The Role of the Family Environment and Computer-Mediated Social Support on Breast Cancer Patients’ Coping Strategies. Journal of Health Communication. 2014 Sep 1;19(9):981–98. [DOI: 10.1080/10810730.2013.864723]
167. Zhou J, Wang G, Zhou T, Fan T. The role of off-topic discussions in online health support groups: insights from a content analysis of an online rectal cancer group. Support Care Cancer. 2020 Jul 1;28(7):3219–26. [DOI: 10.1007/s00520-019-05159-4]
168. Zhu Y, Glowacki EM, Yang Y. A Social Ties-based Approach to Breast Cancer Patients’ Quality of Life: Examining Group Ties and Individual Ties across Offline and Online Settings. Health Commun. 2021 May;36(6):741–51. [DOI: 10.1080/10410236.2020.1712520]
